# Lumbosacral (myelo) meningoceles in dogs, related tethered cord syndrome, and their surgical management: review of the literature and clinical experience

**DOI:** 10.3389/fvets.2025.1510800

**Published:** 2025-03-25

**Authors:** Patrick Roynard, Curtis Wells Dewey

**Affiliations:** ^1^Neurology/Neurosurgery Service, College of Veterinary Medicine, The Ohio State University, Columbus, OH, United States; ^2^Department of Surgery, Long Island Veterinary Specialists, NY, United States; ^3^Elemental Pet Vets, Freeville, NY, United States

**Keywords:** meningocele and meningomyelocele, spina bifida (SB), myelomeningocele (MMC), tethered cord syndrome (TCS), surgery, neural tube defect (NTD), neural tube defect management, Bulldogs

## Abstract

Neural tube defects (NTDs) are a group of congenital malformations characterized by various levels of protrusions of meninges with or without nervous tissue through incomplete osseous coverage (cranium bifidum for the cranial forms and spina bifida for spinal meningoceles/myelomeningoceles [MCs/MMCs]), with associated dorsal midline cutaneous signs. Amongst a confusing vocabulary, spina bifida is both the term most used to refer to NTDs and the most common manifestation of NTDs, with a predilection for the lumbosacral area in screw-tail breeds. With the growing popularity of bulldogs, lumbosacral (LS) MCs/MMCs are increasingly encountered, and small animal practitioners should learn to recognize them. Clinical signs may include urinary and/or fecal incontinence, pelvic limb neurological deficits with bunny hopping (neurolocalization L4-caudal or subset), and cutaneous signs (swirl of hair and dimple); the combination of which is pathognomonic of these disorders in bulldog puppies. Since these malformations often trigger a tethered cord syndrome (TCS), neurological worsening is possible. While historically reported to be somewhat hopeless regarding neurological improvement, isolated case reports, small case series, and personal experience of the author indicates that post-operative improvement is possible. Review of the literature (14 cases) and personal surgical experience (9 cases) retrieved 23 canine cases of LS MC/MMC treated surgically with follow-up. Clinical presentation, diagnostic imaging findings (CT and MRI), and intra- and post-operative findings are discussed in this article, along with a detailed description of the surgical technique. Pelvic limb deficits improve post-surgically in most cases (14/17 [82%] cases with pre-operative deficits and follow-up ≥1 month) albeit sometimes only marginally. Urinary/fecal continence can improve also, although less frequently (10/21 [48%] at 1 month follow-up and 8/21 [38%] at ≥6 months).

## Introduction

**Figure 1:**
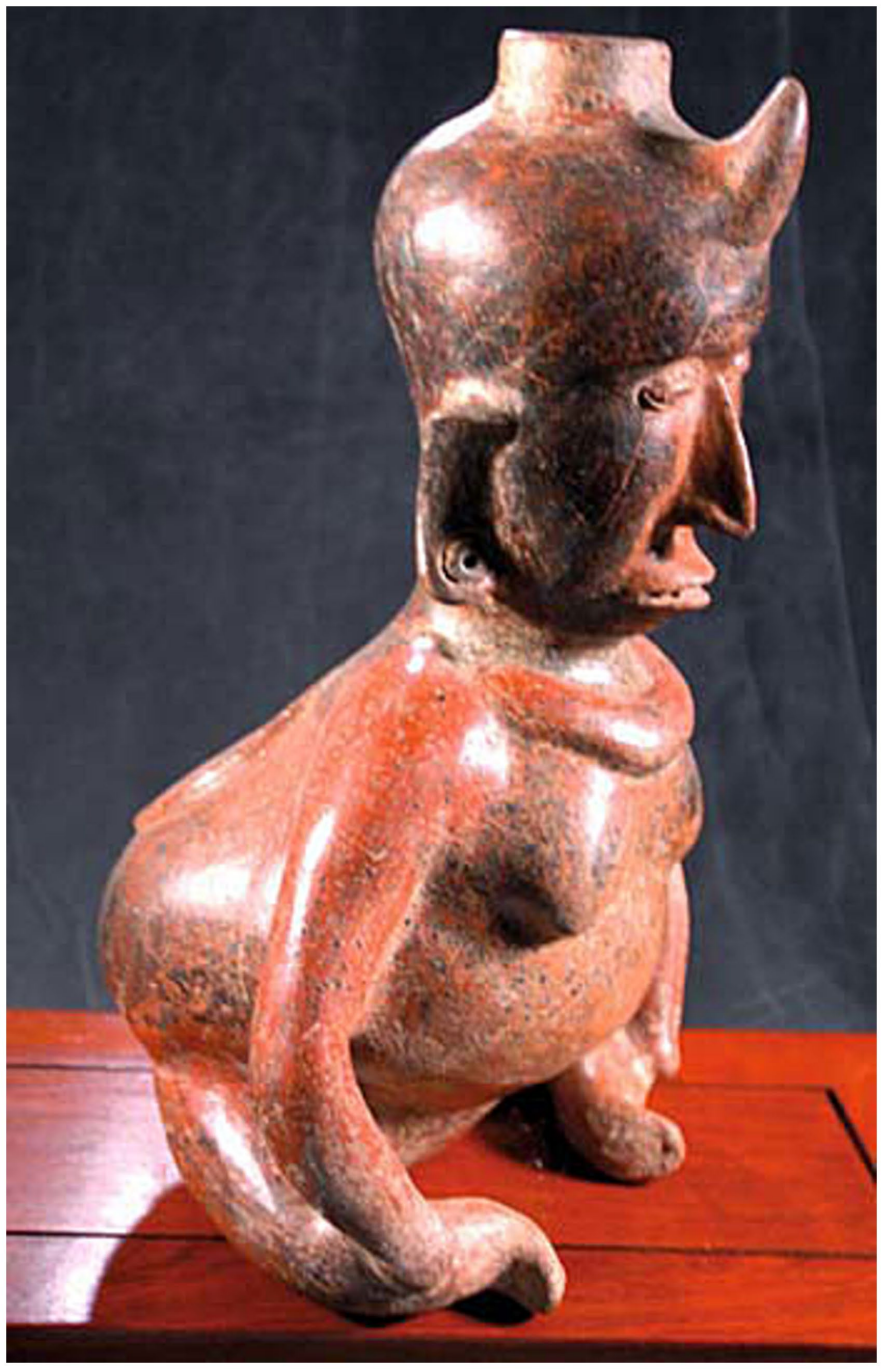
Shaman figure typical of pre-Columbian cultures (Olmec, Nayarit, Colima), West Mexico (circa 200–400 AD). Note the distinctive features of neural tube defects (NTDs) on midline: the “horn,” indicating the shamanic status of the individual, is likely a representation of encephalocele/persistent bregmatic fontanelle associated with this large hydrocephalic head, while a circular medallion is represented on lumbar dorsal midline, where lumbosacral meningoceles/myelomeningoceles (LS MCs/MMCs) show birthmarks or placodes of neuroectodermal tissue. The classic forward-leaning position and severe lumbosacral kyphosis indicate the muscle weakness and vertebral column deformation common with NTDs, while the atrophied legs and their position represent the orthopedic anomalies and paraplegia associated with a LS MMC and severe tethered cord syndrome (TCS) [from Goodrich, J.T. (4), with permission from Springer Nature].

*Meningoceles* and *myelomeningoceles* (MCs/MMCs) are specific types of *Neural Tube Defects* (NTDs), which are characterized by failure of differentiation, formation, and separation of the *neural tube* (NT) from the rest of the ectoderm during neurulation. Dorsal midline cutaneous signs ensue frequently, albeit sometimes subtle, such as the “hair collar” sign in humans with mild cranial forms (1, 2). NTDs have been reported as far back in time as the Neolithic period in humans (3), with representations of such malformations in early civilizations through pottery, sculptures, and other figures (4, 5). As an example, the Olmec (Mesoamerican pre-Columbian civilization) motif of the Were-Jaguar and other figurines representing shamans with specific social status are at least partially inspired by midline defects and physical characteristics of NTDs and MMCs, such as cleft foreheads, cleft/bifid noses, hunchbacks, lumbar kyphosis, sitting positions (due to paralysis of the legs) and circular areas of different skin color in the lower back (representing the cutaneous manifestation of lumbosacral NTDs) (4, 5) (Figure 1).

**Figure 2:**
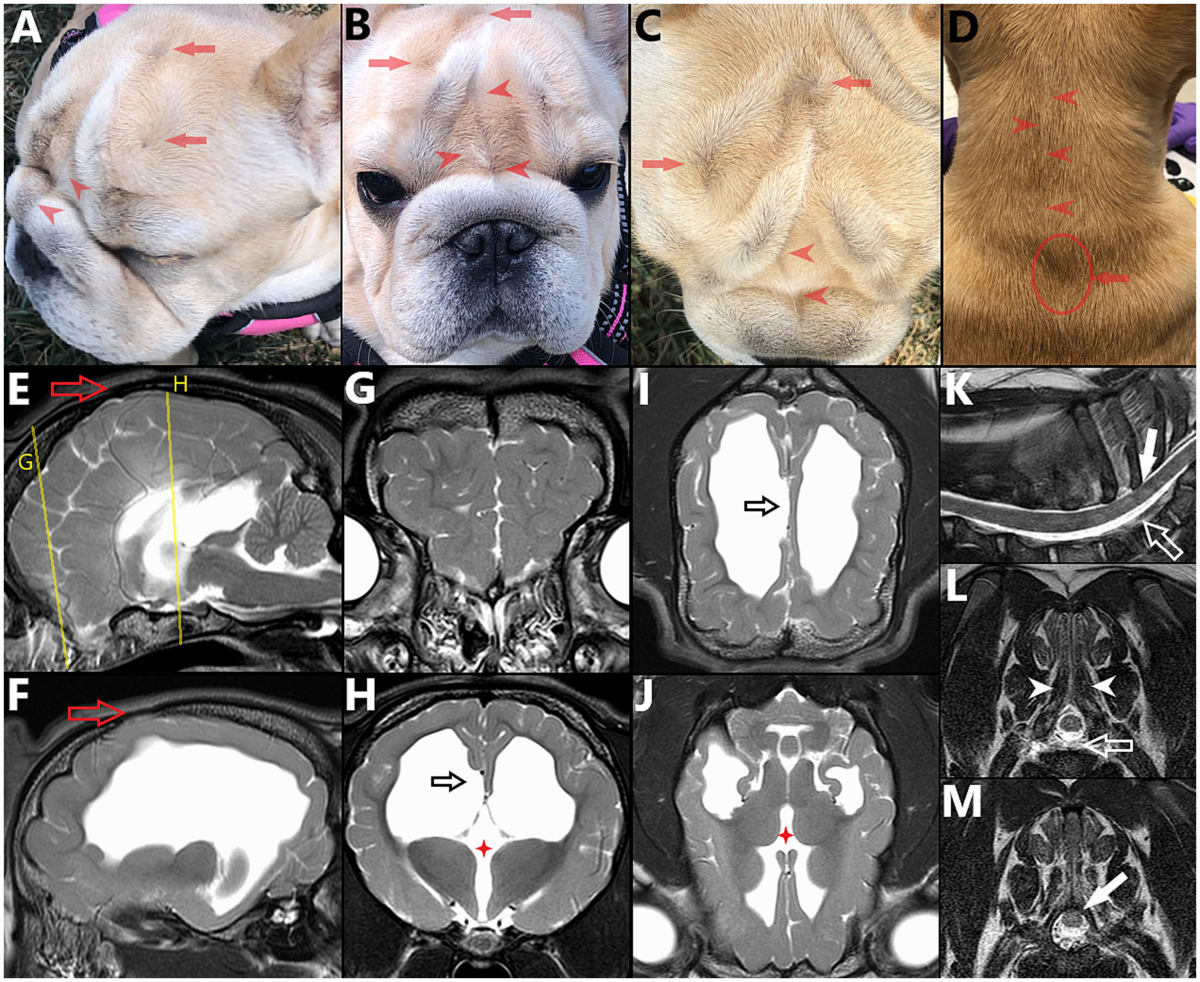
6 month-old female French Bulldog illustrating the concepts of multiple sites of neural tube defects (NTDs) and external/cutaneous signs (visible on dorsal midline) being suggestive of NTDs. **(A)** Lateral, **(B)** frontal, **(C)** dorsal views of the head and **(D)** dorsal view of the neck, with corresponding magnetic resonance images (MRI) vertically aligned. **(E)** Midline and **(F)** para-midline sagittal T2W images of the brain, indicating the level of the transverse T2W images of the **(G)** frontal lobes and **(H)** thalamus, with dorsal T2W images at the level of the **(I)** corpus callosum and **(J)** thalamus. Note the bifid nasal planum (complete cleft in **B**), change of hair implantation with a ridge of hair in the frontal area and on dorsal cervical midline (*red arrowheads* in **A–D**) and swirls of hair over the calvaria (reminiscent of the “hair collar” sign in humans) (*red arrows* in **A–D**), associated with palpable osseous defect (cranium bifidum occultum, *red hollow arrow* in **E,F**). Features of NTD affecting the brain are visible, including hydrocephalus **(F,H–J)**, abnormal gyrification of the frontal lobes **(G)** and failure of interhemispheric midline fusion with agenesis/dysgenesis of midline structures: interthalamic adhesion (*red four points star* in **H,J**) and corpus callosum (*black hollow arrow* in **H,I**, note the abnormal folding of the cingulate gyrus). In the cervical area, under the less pronounced swirl of hair (seen in **D**), **(K)** sagittal and transverse T2W MR images at **(L)** caudal aspect of T1 and **(M)** mid-T2 show further features of NTD/spina bifida: only partially fused spinous process at T1 (*white arrowheads* in **L**), dorsal deviation of the dural sac within the vertebral canal (*white hollow arrow* in **K,L**) and dorsal deviation of the spinal cord within the dural sac (with obliteration of the dorsal subarachnoid space), tucked under the lamina of T2 (*plain white arrow* in **K,M**).

The first extensive, illustrated description of lumbosacral (LS) MC/MMC and surgical correction, along with the first use of the term *spina bifida* (SB), must be credited to the Dutch surgeon Nicolaes Tulp (1593–1674) in his book *Observationes medicae* (6). In humans, MC/MMC is a clear indication for surgery, with the current standard of care being in-utero surgery since the findings of the MOMS trial in 2011 showing clear benefits of prenatal vs. postnatal surgical correction (7). Although reports of MCs/MMCs exist in the veterinary literature for more than a century, they are most commonly descriptive, and there is currently a lack of long-term clinical studies evaluating surgical or medical management in companion animals. NTDs such as MCs/MMCs are most often encountered in the LS area in screw-tail breeds (e.g., Bulldogs for dogs, Manx for cats), but have also been reported in other breeds and other areas along dorsal midline of the neuraxis (e.g., head, cervical and thoracolumbar vertebral column) (8–11) (Figure 2). For LS MCs/MMCs, the classical presentation is urinary and fecal incontinence with neurological deficits of variable severity affecting the pelvic limbs in a young/immature animal. Although historically reported to be static clinically, these disorders can be progressive if associated with *tethered cord syndrome* (TCS) and the neurological deficits observed can worsen in the absence of surgical correction. As Bulldogs have become some of the most popular breeds and owners reluctant to euthanize, patients with LS MCs/MMCs are seen more frequently in need of a therapeutic option.

Through previous work with Bulldog rescue organizations, the authors were involved with multiple cases of LS MCs/MMCs, including 9 cases treated surgically, some previously reported (12). In the English veterinary literature, the authors could retrieve 14 canine cases of LS MC/MMC treated surgically with information regarding clinical signs and outcome (albeit occasionally incomplete) (13–19). Two other Bulldogs were treated surgically for LS MC/MMC with overall similar technique, but the procedure included intra-operative application of allogeneic placenta-derived mesenchymal stem/stromal cells at the surgical site (20), so they are not included in the results of this review. This article attempts to clarify the terminology and concepts of NTDs before reviewing the literature and personal experience of the authors with surgical management of canine LS MCs/MMCs.

## Terminology

*“Ce que l’on conçoit bien s’énonce clairement…”* (what we understand well we explain clearly [Nicolas Boileau 1636–1711]).

**Figure 3:**
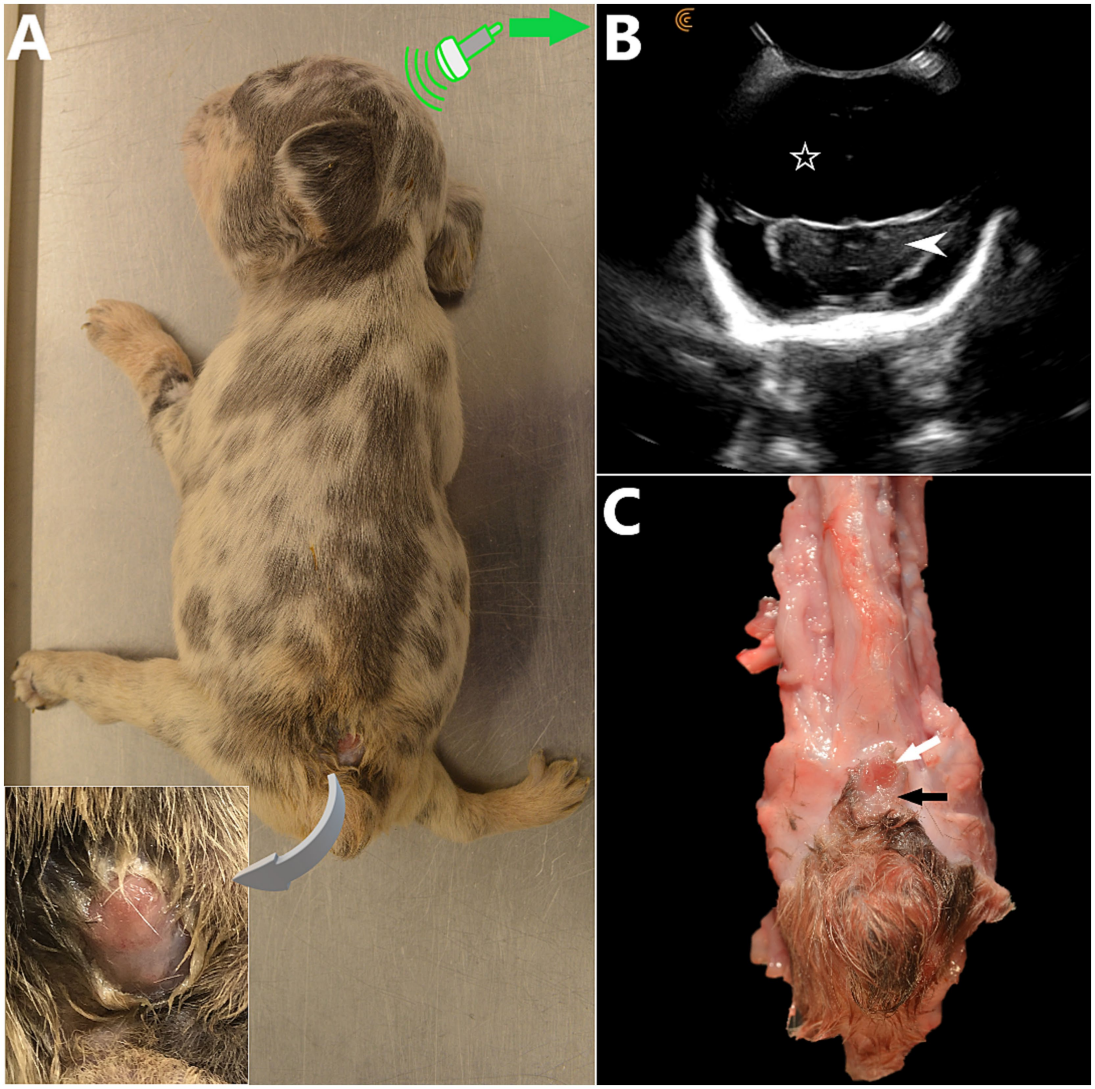
**(A)** 5 week-old female French Bulldog with severe, open lumbosacral myelomeningocele (LS MMC) and spina bifida (SB) at S1-S2 with protrusion of a placode of abnormal, non-neurulated neuroectodermal tissue in the LS area (similar to human MMCs). Two other dogs from the litter were euthanized due to severe malformations at delivery (C-section). This puppy had severe neurological deficits and orthopedic malformations (non-ambulatory tetraparesis markedly worse in the malformed pelvic limbs), dome-shaped head and persistent bregmatic fontanelle. The placode of non-neurulated neuroectodermal tissue is covered with a layer of dysplastic epidermal tissue, continuous with it and ulcerated segmentally. CSF leakage was present at this site (*insert*). **(B)** Transfontanelle brain sonogram, transverse view at the level of the diencephalon (*white arrowhead*) showing hydrocephalus with severely enlarged anechoic lateral ventricles (*white hollow star*). **(C)** Anatomical specimen, spinal cord removed (cranial aspect at the top). The non-neurulated spinal cord is visible at skin level as 2 unfused hemicords (*white arrow*), bordered by an alopecic area of abnormal tissue (pial layer of the meninges having failed to close and fused laterally to the epidermis) (*black arrow*). *(A and C courtesy of Dr. Kate Sarkan).*

The terminology used in the medical literature on NTDs is confusing to say the least. Terms employed are somewhat redundant (e.g., MCs/MMCs are exemples of NTDs, myelodysplasia, spinal dysraphisms and also imply the presence of spina bifida) and confusing due to the subtlety in their differences or lack thereof (e.g., myelodysplasia defined as “any malformation of the spinal cord owing to abnormal interaction of the notochord, paraxial mesoderm, and neural plate during neurulation” and dysraphism defined as “failure of neural folds to oppose and close, resulting in failed neural tube closure”) (21–23). Proper terminology is also inconsistently or inaccurately applied (the terms NTDs, myelodysplasia, spinal dysraphism and spina bifida are often used interchangeably, while stricter definitions differentiate them; also see Discussion on dermoid sinus in the veterinary literature), and with wide interspecies meaning variation (e.g., while in humans the term MMC is used for NTDs with protrusion to the surface of a placode of abnormal neuroectodermal tissue (Figure 3), the protruded meninges are most commonly covered by the skin and various levels of fibrous tissue in dogs – more similar to human lipomyelomeningoceles which are skin covered NTDs). Table 1 presents a classification and the terminology associated with NTDs, along with the associated bone defects (cranial and spinal malformations are presented, respectively, on the left half and right half of the table, at similar level of severity). Spina bifida, although referring strictly speaking only to the bony anomaly, remains the term most used to designate these NTDs, likely due to historical ability to diagnose it on radiographs. Naming the underlying CNS malformation is more descriptive, useful clinically (spina bifida is not, as the bony malformation is clinically irrelevant from a neurological perspective) and should be preferred when discussing these NTDs.

**Table 1:** Classification and terminology of neural tube defects (NTDs).

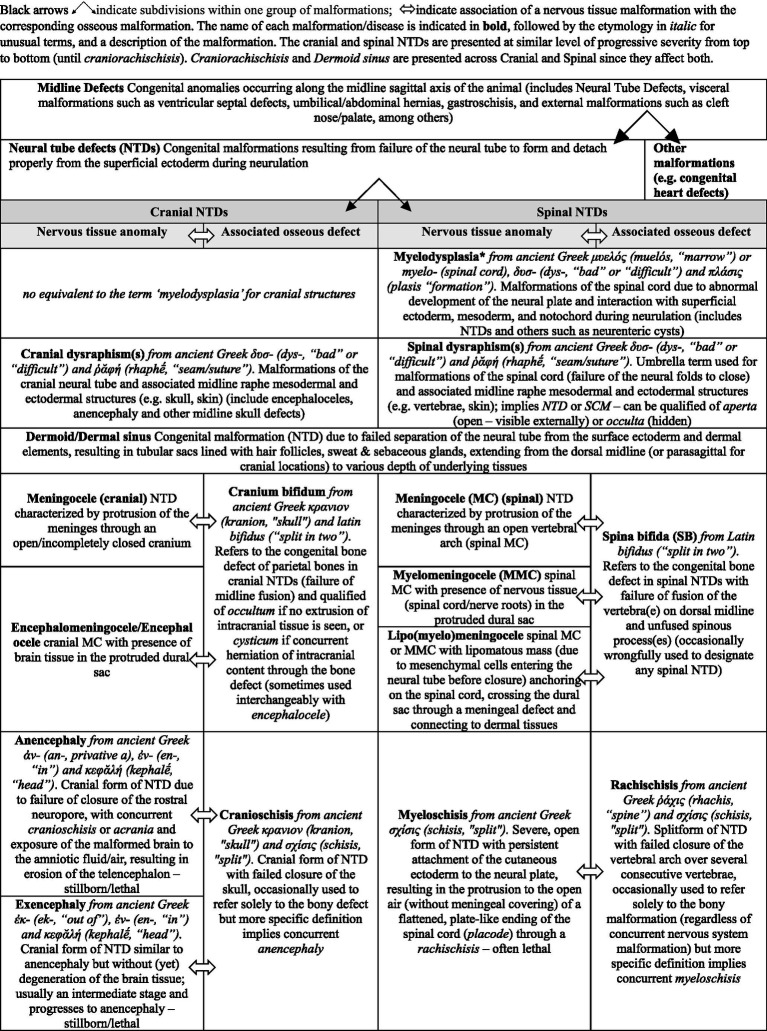	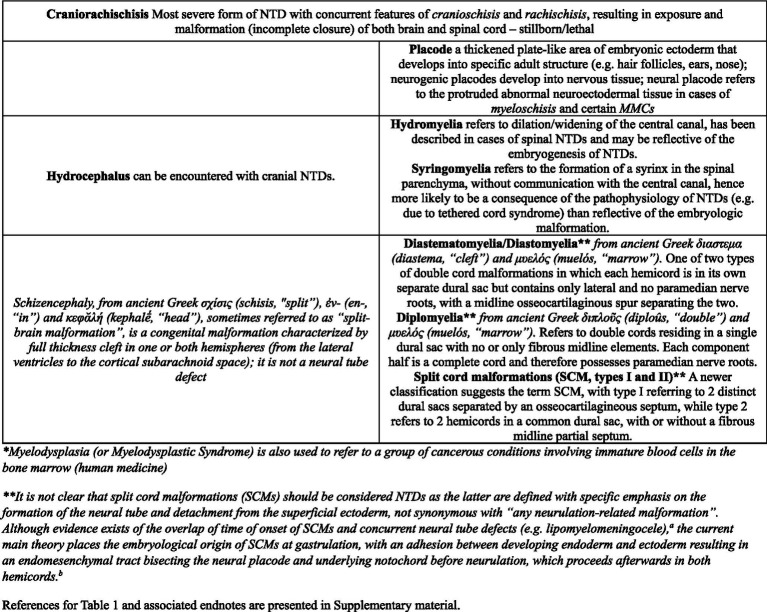

## Etiology (pathogenesis) and pathophysiology

### Embryology - neurulation

**Figure 4:**
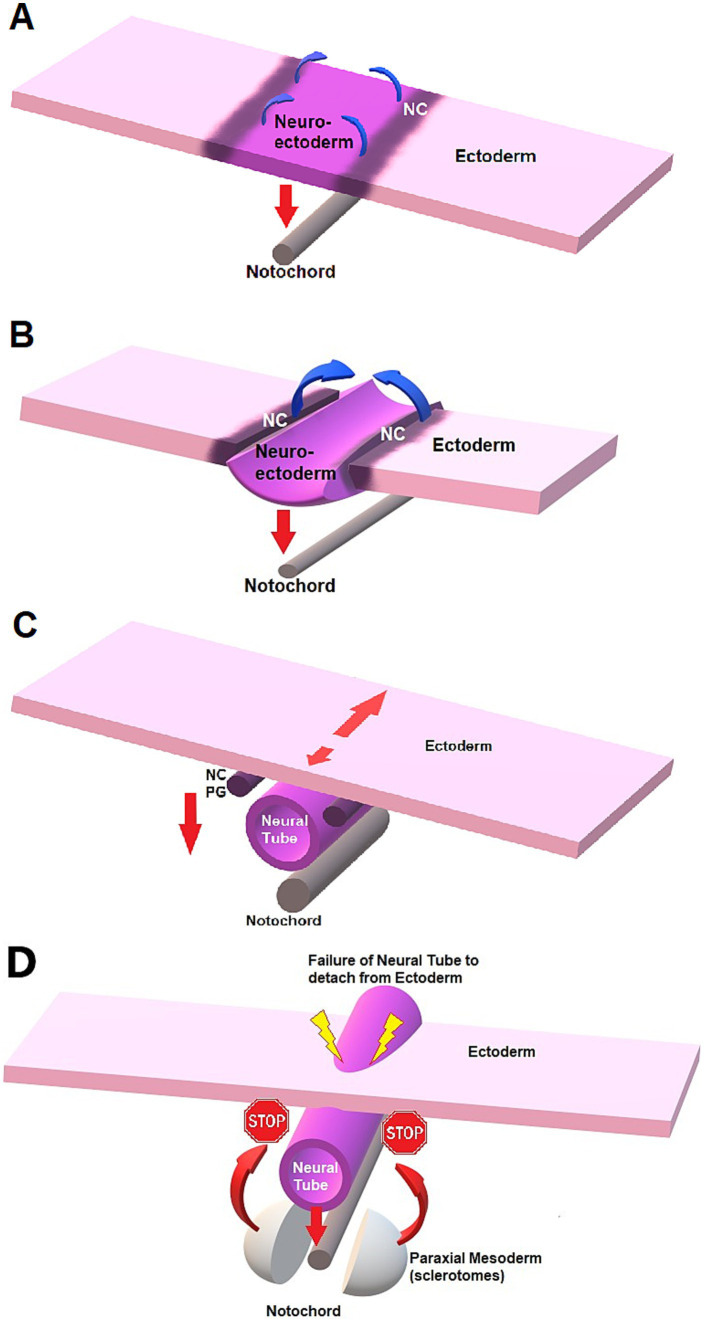
Schematic representation of neurulation and failure to detach in NTDs. **(A)** Neural plate stage. The notochord exerts an influence on the neural plate (neuro-ectoderm) for formation of the neural tube through signaling molecules such as sonic hedgehog (*red down arrow*). The ventral aspect of the neural plate bends and is attracted to the notochord ventrally, creating a midline sulcus while the neural crests (NC) cells elevate (*curved blue arrow*). **(B)** Neural groove. The lateral margins of the neural plate elevate and create the neural folds located at the junction neural plate-ectoderm longitudinally. The folds then get closer to midline until they fuse dorsally, creating a tubular structure: the neural tube. **(C)** As the neural tube detaches from the dorsal, superficial ectoderm to migrate ventrally towards the notochord, the NC cells detach and migrate ventro-laterally on each side. The NC cells will form the dorsal spinal cord and spinal ganglia, after forming the primary ganglia (PG). The number of sites of initial fusion varies per species, and closure proceeds in a zipper-like fashion either unidirectionally or bidirectionally along the long axis, also depending on the species. Neurulation can hence continue cephalad (*short red arrow*) and caudad (*long red arrow*) while the neural tube “migrates” ventrally under the influence of the notochord (*vertical down arrow*). **(D)** NTD and failure of the neural tube to detach from ectoderm, preventing mesodermal structures to fuse on dorsal midline.

Embryologically, the spinal cord originates from the neurectoderm, after formation of the neural tube and separation from the remaining ectoderm during neurulation (24). During embryogenesis, the development of the nervous system occurs through *neurulation*, which starts by the end of *gastrulation*, when the three germinal/embryonic layers are formed (*ectoderm*, *mesoderm*, and *endoderm*). Neurulation proceeds as *primary neurulation* for the anterior and largest part of the central nervous system, and *secondary neurulation* for the most caudal part (sacral and caudal spinal segments). The formation of the neural tube occurs through infolding of the neural plate under the effect of the notochord ventrally, initial closure at (a) given point(s) of the neuraxis, and propagation in a zipper-like fashion along the long axis (Figure 4). Most commonly initial closure(s) occur(s) at some level of the brain or cervical area with a short rostral propagation leading to the *cranial neuropore* (first opening to close) and a longer caudal propagation leading to the *caudal neuropore*. Closure of the caudal neuropore marks the end of primary neurulation. Secondary neurulation is species-specific and occurs at the caudal bud/tail bud, forming the last sacral and caudal spinal segments, along with mesodermal derivatives.

**Figure 5:**
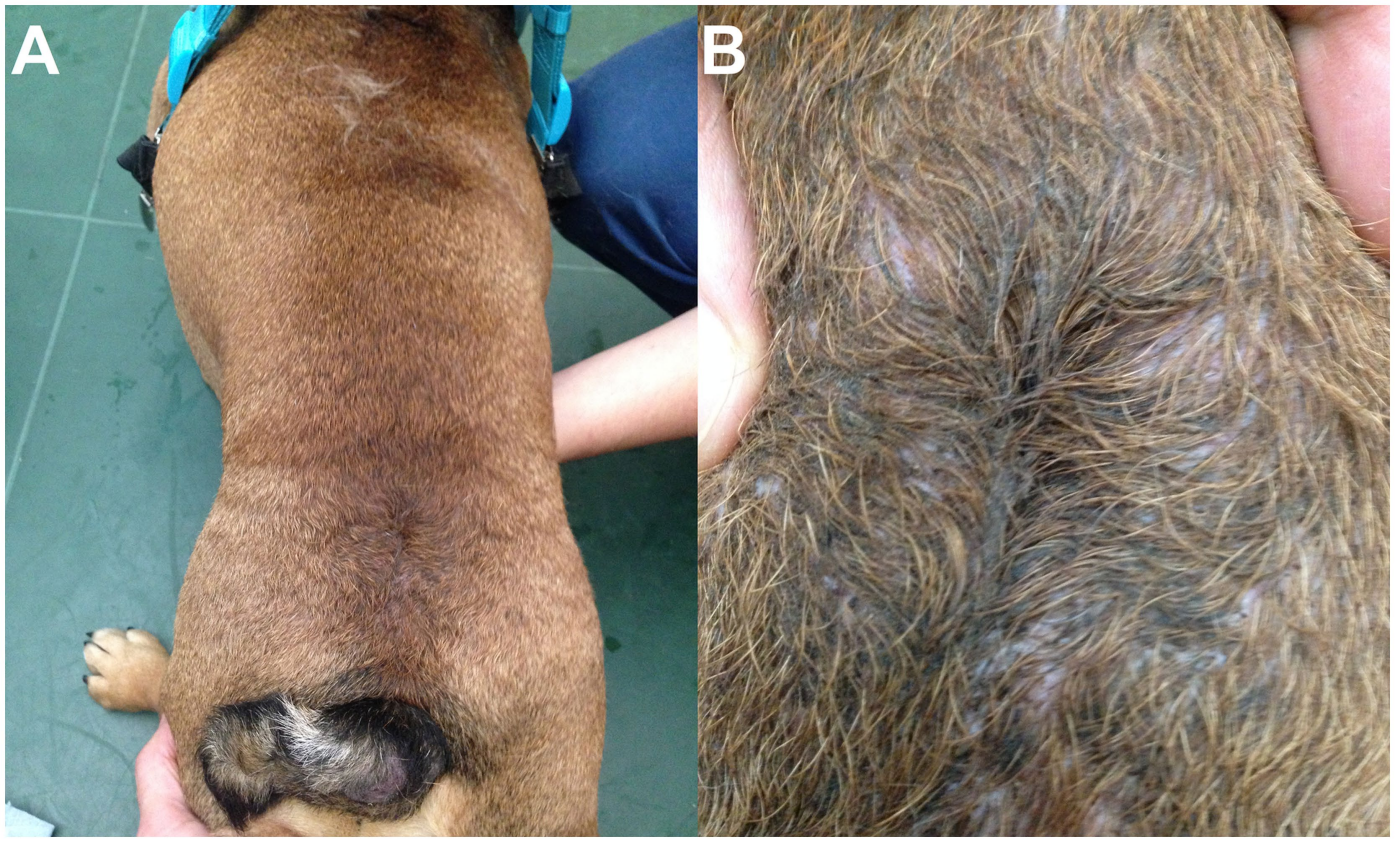
1.5-year-old male neutered English Bulldog mix with open lumbosacral (LS) meningocele (MC). **(A)** A swirl of hair and dimple are visible in the dorsal LS area. **(B)** Close-up view showing the CSF leakage and moist aspect of the skin, at the distal, open end of the MC.

*Neural tube defects (NTDs)* refer to different congenital anomalies characterized by failure of closure of the neural tube during neurulation. Failure of closure of the rostral neuropore can lead to anencephaly, a condition characterized by lack of formation of the cerebral hemispheres (brainstem and cerebellum are abnormal, but present) and protrusion of the malformed brain through an opening in the cranial vault having failed to close (*cranioschisis*), often fatal and associated with stillbirth. Less severe forms of cranial NTDs are “closed” and compatible with life, such as encephaloceles, characterized by protrusion of the meninges with (*meningoencephalocele*) or without (cranial *meningocele*) brain tissue through a skull defect (*cranium bifidum*) that is covered with skin. Milder forms of human cranial NTDs may be suspected based on dorsal midline cutaneous signs, marks of NTDs on the ectoderm (e.g., the “hair collar sign,” a ring of darker, coarser hair surrounding a congenital scalp lesion), and similar semiology can be applied to canine patients (see Figure 2). In NTDs affecting the spinal cord, the failure of the neural tube to detach from the ectoderm prevents the mesodermal structures such as the sclerotomes from surrounding the nervous tissue and prevent fusion of said bilaterally paired hemi-structures on dorsal midline (see Figure 4D). This results in dorsally opened vertebrae with 2 dorsolateral hemi-laminae and 2 unfused spinous processes, hence the term *spina bifida* (from Latin *bifidus* which means split in half by a longitudinal cleft). When the caudal neuropore is affected, this results in LS MCs/MMCs, with various levels of protrusions of the meninges +/− malformed nervous system through an open vertebral arch (potentially with protrusion at the air of malformed nervous tissue itself) in association with lack of complete midline fusion of overlying mesodermal structures (e.g., muscles, vertebral spinous process) in the lumbosacral area (see Figure 3). Open MC/MMC is a term used for cases with communication of the meninges with the skin and leakage of CSF through the integument (Figure 5).

MCs/MMCs are responsible for neurological deficits due to the anatomical malformation itself, possible lack of normal neural development (due to lack of closure of the neural tube – see Discussion), CSF leakage triggering electrolyte abnormalities or favoring ascending infectious meningomyelitis (for open MCs/MMCs) and possible tethered cord syndrome (TCS).

### Tethered cord syndrome: etiology and clinical relevance in LS MCs/MMCs

**Figure 6:**
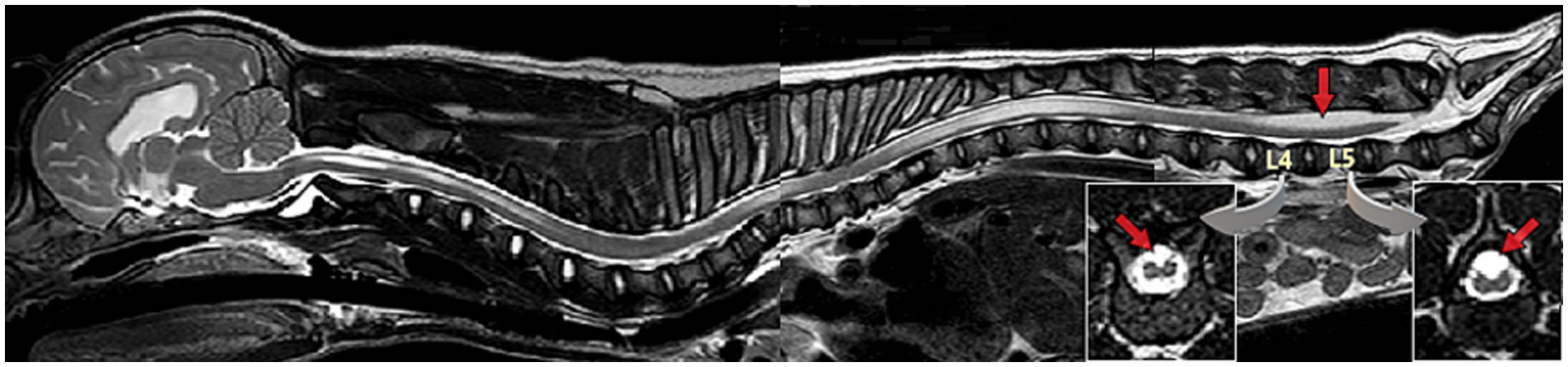
Sagittal T2W magnetic resonance (MR) image whole spine & brain (mobiview) of a 3-month-old male French Bulldog/pug mix with spina bifida (SB) at L7 and lipomyelomeningocele with resulting tethered cord syndrome (TCS). Note the area of abnormal unfused/split cord over the vertebral bodies of L4-L5, more conspicuous on transverse images (*inserts*) (see Diagnosis/Diagnostic Imaging for further discussion), the syringomyelia in the thoracolumbar and cervical spinal cord, and the caudal cerebellar indentation.

In early embryogenesis, the spinal cord occupies the entire length of the vertebral canal and the origin of each pair of spinal nerve roots (dorsal and ventral) along the spinal cord aligns with their exit point of the vertebral column at each respective intervertebral foramen. During embryologic life and post-natal growth until adult size is reached, the spinal cord growth slows down while the rest of the body (e.g., vertebral column) continues to extend longitudinally. This differential in growth in the longitudinal axis between the nervous tissue and the rest of the vertebral column is responsible for a relative ascending movement of the conus medullaris cranially within the vertebral column during growth. Consequently, in an adult mid-size dog, the end of the spinal cord/conus medullaris reaches approximately the sixth or seventh lumbar vertebra, with further cranial locations reported in large dogs (25–27). Hence, the most caudally located spinal nerve roots (lumbar, sacral and caudal) elongate to reach their now relatively more caudal exit point of the vertebral column, creating the *cauda equina*. This ascending movement is allowed by elongation and the elastic properties of the *filum terminale*, a ligamentous structure formed during secondary neurulation and connecting the conus medullaris to the distal sacrum or first caudal vertebra. This differential in longitudinal growth between spinal cord and vertebral column, along with the anchor point of the rostral end of the neuraxis (the brain encompassed in the skull), explain the relative tension/traction put on the conus medullaris if an added anchor point is also present caudally. In NTDs in general and LS MCs/MMCs specifically, the failure of detachment of the neural tube from the ectoderm acts as that anchor point. This progressive and repetitive or sustained traction is responsible for decreased blood flow and decreased oxidative metabolism in the conus medullaris, and potentially more cranial structures (rest of the spinal cord), with syringomyelia as possible consequence (Figure 6).

*Tethered cord syndrome* (TCS) refers to a constellation of neurological deficits, dermatological, gastrointestinal, urological, and occasionally orthopedic signs, due to abnormal traction on the conus medullaris and spinal cord. This occurs secondary to mechanical tethering from abnormal anatomy (e.g., cases of malformation such as LS MCs/MMCs in bulldogs) or from abnormally inelastic tissue within the filum terminale (28–30). Occult tethered cord syndrome (OTCS) refers to TCS with tethering of the spinal cord by the filum terminale, in the absence of diagnostic imaging abnormalities on static images (including conus medullaris in normal position), and diagnosis may require dynamic views (i.e., comparative images obtained with LS area in flexed, neutral, and extended position) (29, 30). In diagnostic imaging, TCS has been described as a conus medullaris with abnormally caudal position on static images or lacking craniocaudal mobility within the vertebral canal on dynamic views, or as a dorsal displacement of the spinal cord being visualized within or outside the vertebral canal, and can be encountered in other areas of the spinal cord (e.g., thoracic) (28, 29, 31). Cases of TCS are not always associated with the most severe amount of mechanical traction/restriction, but the authors have diagnosed several cases of NTDs with TCS having developed syringohydromyelia in thoracolumbar or even cervical spinal cord on MRI (see Figure 6; see Diagnosis/Diagnostic Imaging/MRI).

## Diagnosis

### Clinical

#### Population - signalment

Prior descriptive publications on LS MCs/MMCs report an over-representation of screw tail breeds and Bulldogs specifically (11, 21, 32), as found in the 14 literature cases treated surgically with 12/14 dogs being Bulldogs (8 French Bulldogs and 4 English). The remaining 2 dogs in the literature were a German shepherd dog and a Yorkshire terrier (13–19). All dogs in our cohort were Bulldogs, 6/9 English Bulldogs (including 1 English Bulldog x Pitbull mix) and 3/9 French Bulldogs (including 1 French Bulldog x Pug mix).

Clinical signs are present from birth, specifically the physical abnormality(ies) related to the NTD (see Physical examination below); neurological deficits are also present from birth, although they may worsen with time in cases of TCS and the paresis and incontinence may not be confirmed before several weeks old, when pups in the litter start exploring otherwise. In our cohort, the age of dogs at time of surgery was between 3 and 6 months old with an average of 4.2 months old. The literature cases were between 7.5 weeks and 2 years old, for an average of 5.2 months old, in the 11/14 where age was available (only 1/11 dogs was older than 5 months old – the one who was 2 years old; for a corrected average age at surgery of 3.3 months in 10 dogs if that dog was excluded).

Sex repartition might show a slight male predilection, with 6 males and 3 females in our cohort and 7 males for 4 females in the literature (3/14 sex unspecified). Previous non-interventional studies reported, respectively, 2/3 males (one unspecified) (10) and 18 males for 14 females (11).

#### Physical examination

**Figure 7:**
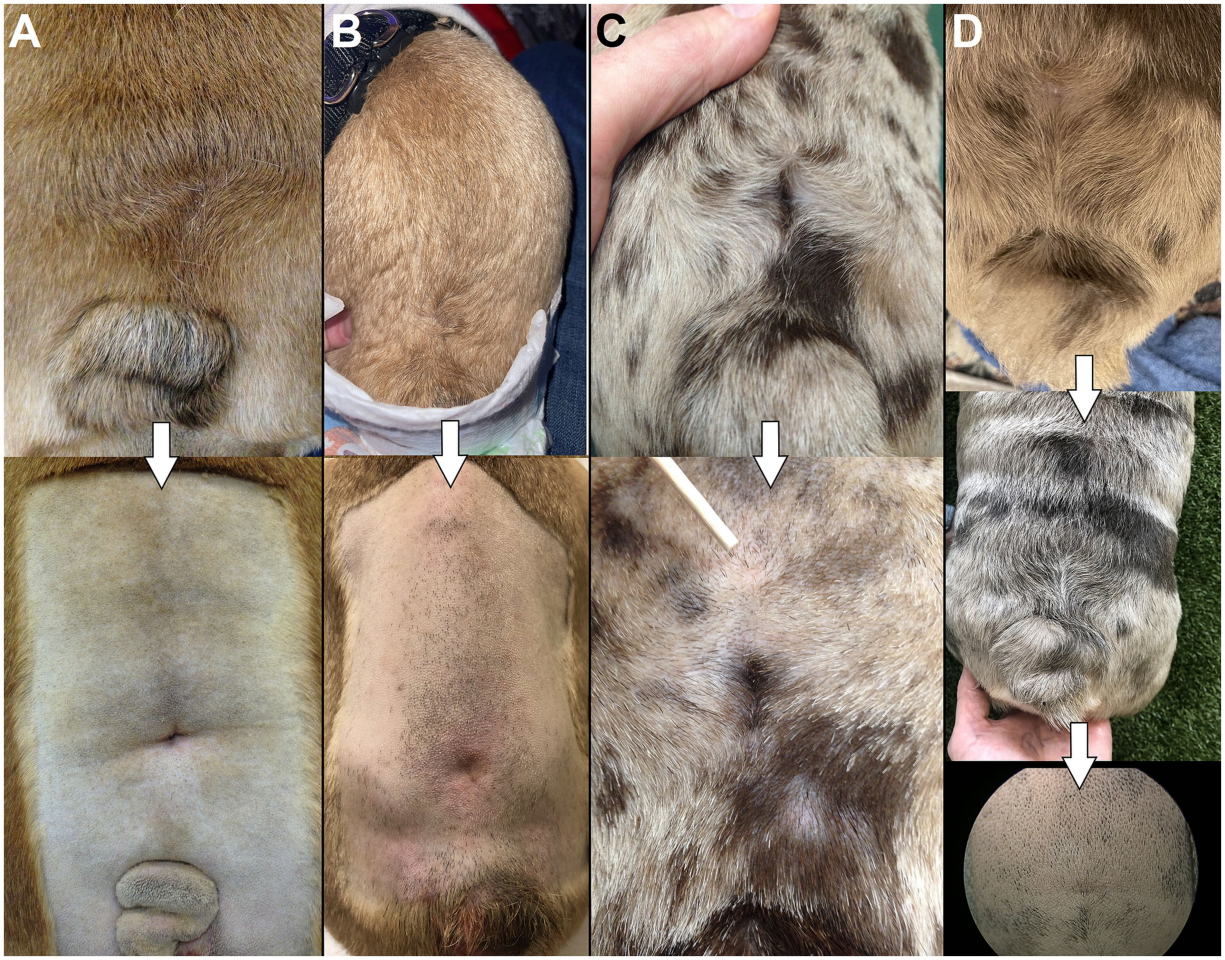
“Swirls and dimples”. **(A–D)** Examples of the change in hair implantation (“swirl”) and depression in the skin/muscles (“dimple”) at the site of LS MC/MMC in 4 different bulldog puppies included in our surgical cohort. Top images show dorsal view, bottom images show magnification after being clipped for surgery.

On visual examination, patients with LS MCs/MMCs are often juvenile and present an abnormal LS area with various levels of severity. The most common findings are cutaneous signs with a change in hair implantation (“swirl,” also reported as streaming of hair) (11) and a depression in the muscles and soft tissues (“dimple”), both on lumbosacral dorsal midline (Figure 7). This was noted in 9/9 authors’ cases and reported in 10/14 cases in the literature (in 4 cases the information was missing). For cases of open MC/MMC, the leakage might not be obvious while covered in hair as a crust may form (15); in some cases the area is visibly moist (see Figure 5). This was reported in 1/14 cases in the literature (15) and none in our surgical group. The most severe cases may show a “placode” of neuro-ectodermal tissue as reported in humans, although this is rare in the authors’ experience (see Figure 3). In our experience, cutaneous signs suggestive of NTDs are also commonly encountered in the cervical and cranial thoracic areas.

#### Concurrent malformations

**Figure 8:**
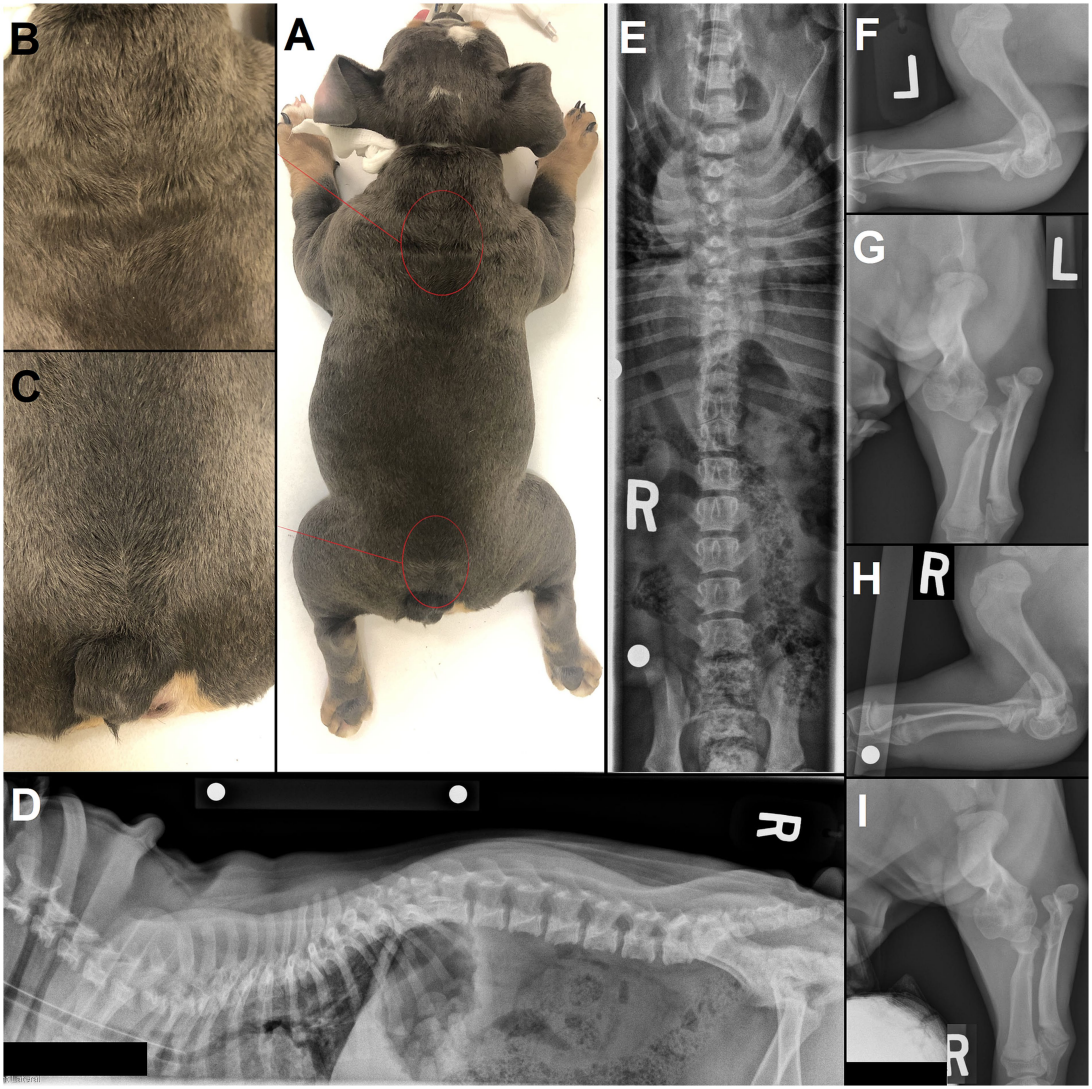
4 month-old male English Bulldog with several sites of neural tube defects (NTDs) and concurrent malformations. **(A)** Several areas on dorsal midline show a swirl of hair, most noticeable in the **(B)** upper thoracic and **(C)** lumbosacral area. **(D)** Lateral and **(E)** ventrodorsal radiographs of the vertebral column show an abnormally large spinous process at T1, a bifid, partially fused spinous process at T7 and lack of spinous process at L7 and S1, along with further vertebral malformations (e.g., hemivertebrae and wedge-shaped vertebrae in the thoracic vertebral column, butterfly vertebra with missing right rib at T13). **(F,H)** Lateral and **(G,I)** dorsal radiographs of the left and right elbows show marked vertebral malformations of the thoracic limbs, with severe bilateral elbow dysplasia and luxation.

As a rule of thumb with congenital malformations, the earlier in embryology the malformation occurred, the more likely it is to have triggered further anomalies in the embryological development (33, 34). Since NTDs occur early on (neurulation), concurrent malformations – not limited to the nervous system – occur as expected and may be extensive, such as caudal regression syndrome, and/ or include a lethal character (35). Cryptorchidism was present in 2/6 males in our population and 1/7 in the literature (15). Concurrent severe orthopedic issues (joint deformities, severe hip dysplasia) were present in 3/7 cases where that information was available (diagnostic imaging of the pelvic limbs performed or physical examination documenting complete orthopedic examination; in 2/9 cases that information could not be retrieved). Although not included in the surgically treated cohort (owners’ decision), the authors have also encountered dogs with external and/or radiographic signs of multiple concurrent NTDs and other malformations, such as severe limbs deformities (see Figures 3, 8), as reported in the literature (10, 11, 36, 37).

#### Neurological examination

Neurological deficits reflect the anatomical malformation +/− TCS and most often affect the sacrocaudal nerves with all 9/9 of our patients localizing to an L4-Cd myeloradiculoneuropathy (4/9 showed no deficits of the femoral nerve for a more specific L7-Cd neurolocalization, 2 of which also showed normal withdrawal reflex [decreased in the other 7/9] with normal gastrocnemius reflex in 1 of the 2). Urinary and/or fecal incontinence were reported in all cases (9/9 of our cases and 14/14 in the literature), with some dogs having never voluntarily voided or postured to eliminate to the owner’s knowledge. Involvement of the sacral nerves is the rule with abnormal perineal reflex (decreased in 3/9 and absent in 6/9) and anal tone (decreased 3/9, absent 6/9) in all our cases. This was not always retrievable in literature cases but only 1/14 reported intact perineal reflex and anal tone – it is noticeable that this dog was the most caudally affected with the MMC affecting the sacrum and first caudal vertebrae (15). Hypoalgesia or analgesia of the perineal area and/or tail base was also present in 7/9 of our cases (normal sensation in the other 2) and in the 4/14 literature cases (13, 15–17) where information on perineal sensation was reported (no information available in 10/14 literature cases) (14, 18, 19). In the authors’ experience, hypoalgesia of the caudal aspect of the pelvic limbs is frequent, although this was not systematically assessed in our first cases. Postural reactions deficits (e.g., proprioceptive placing, hopping) in the pelvic limbs were ubiquitous in our cohort (9/9), although sometimes only mild. Pelvic limbs gait deficits (paresis, ataxia) are frequent in our experience, present in 8/9 cases, albeit only minimal to mild in 5/8 and moderate to severe in 3/8 (one of the 9 dogs presented delayed proprioceptive placing in both pelvic limbs as sole deficits, with no obvious ataxia nor paresis seen on examination). Literature cases account for 10/14 dogs with PLs deficits before surgery varying from minimal to non-ambulatory paraparesis, although specific neurolocalization was not reported consistently (13–19). Another common gait anomaly encountered with NTDs is bunny hoping, present in most patients (7/9 puppies in our cohort) with varying severity and types of gaits affected (walk vs. trot vs. gallop), although infrequently reported on in the literature (13–19). As expected with congenital malformations, pain is not a common feature and although the authors have encountered some dogs showing mild discomfort on LS palpation, this was not a predominant feature in any of the dogs we treated surgically. It was reported in only one dog in the literature (18). Perhaps more surprisingly, signs of neuropathic pain (e.g., dysesthesia, paresthesia) are not frequently identified in these patients, and were not reported in our group nor in any of the 14 literature surgical cases.

In our experience, clinical signs of TCS with progressively worsening neurological deficits in an ascending pattern was frequent (7/9 puppies), albeit mild and seemingly finite (i.e., last recheck neurological examination was static) in most (5/7). For 2/7 cases with TCS, the last neurological examination before surgery reported minimal/mild worsening, so it could not be ascertained that clinical signs of TCS had stopped worsening. Most of the dogs included in our surgical cohort were monitored for a few weeks prior to surgical intervention, allowing for repeated neurological examination. As reported by previous authors (38), it is our impression that clinical TCS with worsening is frequent in this population at a young age (i.e., between 4 weeks and 16 weeks old), although this was not commonly examined/reported on in the literature. Three single surgical cases reported progression of neurological signs or late onset of signs consistent with worsening TCS (13, 17, 18), while no details specifying progression or lack thereof were found for the other 11 cases (14–16, 19). A common manifestation of progressive TCS would be dogs presenting with initially only sacrocaudal deficits (absent perineal reflex & anal tone, incontinence) showing sciatic nerve deficits at first recheck (e.g., decreased withdrawal in the pelvic limbs at the hock), and potentially femoral nerve deficits (e.g., decreased patellar reflex, weakness at the stifle) later.

### Diagnostic imaging

#### Radiographs

**Figure 9:**
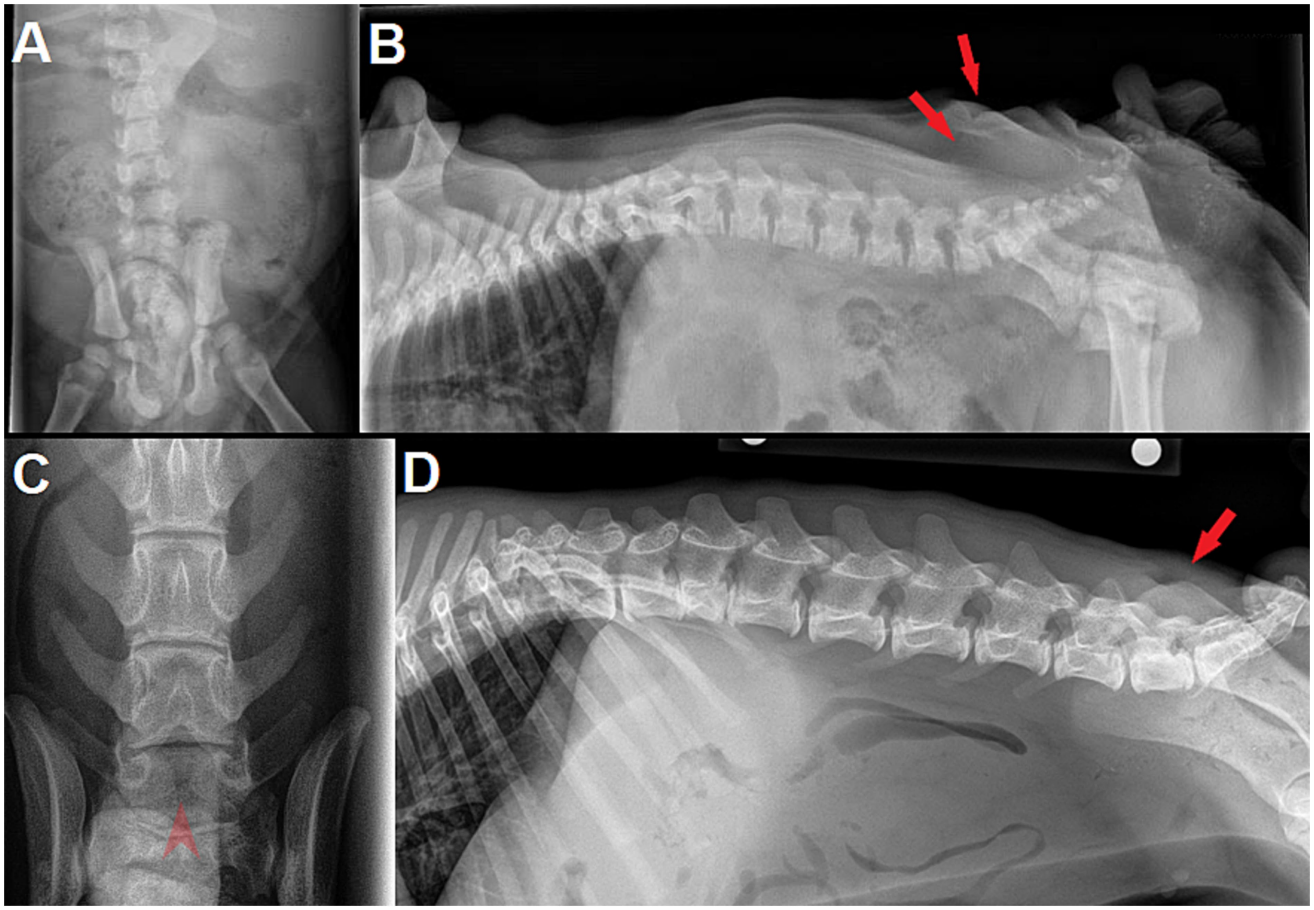
**(A)** Dorsoventral (DV) and **(B)** lateral radiographs of a 9 week-old male English Bulldog with myelomeningocele (MMC) and spina bifida (SB) at L6 (dog D in Figure 7). Note the abnormally small silhouette of the spinous process at L6 on lateral view, along with the skin fold and a mild radiolucent line at the level of the dimple (*red arrows*). Super-imposition of fecal material in the colon prevents proper visualization on DV view. **(C)** Dorsoventral and **(D)** lateral radiographs of a 5 month-old male French Bulldog with MMC and SB at L8 and S1. Note the clear visualization of 2 unfused, hemi-spinous processes at L8 on DV view (*red arrowhead*), while the skin dimple is not as obvious on lateral view (*red arrow*).

The osseous part of the malformation (spina bifida) and other concurrent vertebral malformations can usually be seen on routine radiographs, although proper placement is paramount to diagnostic quality. Dorsoventral/ventrodorsal view of the lumbosacral vertebral column is diagnostic for spina bifida if a bifid spinous process can be identified, although technical factors can complicate interpretation (e.g., proper exposition, super-imposition of stools in colon in cases with megacolon) (see Figures 8, 9). It can be difficult to commit to a radiographic diagnosis if the only finding is the absence of a clear spinous process on VD/DV views, without identification of an abnormal, bifid one. Lateral radiographs may also occasionally help identify the skin dimple (see Figure 9). As reported in Bulldogs previously, concurrent vertebral malformations at other sites (e.g., wedge-shaped vertebrae, hemivertebrae, butterfly vertebrae, other sites of SB) are frequent in the authors experience and were found in 9/9 dogs (see Figure 9).

#### Computed tomography

##### Location of the main spina bifida site

**Figure 10:**
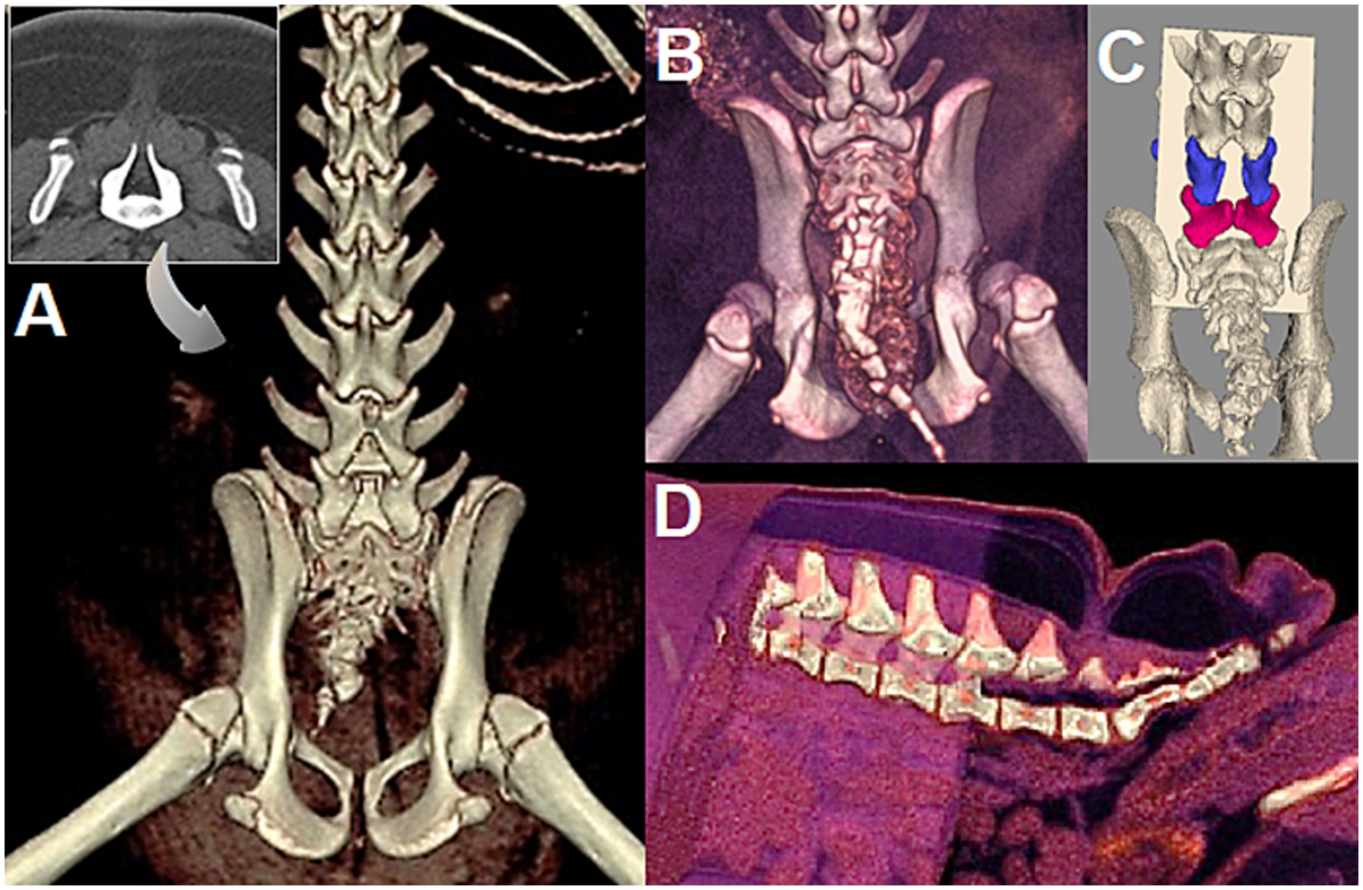
**(A)** 5-month-old female English bulldog (Dog A in Figure 7), CT 3D reconstruction and 2D transverse view *(insert)* at site of spina bifida (SB) at L7. Note the abnormal spinous process/lamina at L6 and SB in the caudal vertebrae. **(B)** 5-month-old female English bulldog (Dog C in Figure 7), CT 3D reconstruction with SB at S1. Note the SB of caudal vertebrae and severe hip dysplasia/incongruency. **(C)** 3.5 month old male English Bulldog (Dog D in Figure 7, radiographs in Figures 9A,B), CT 3D reconstruction. Note the SB at L6 *(blue)*, L7 *(magenta)*, and the slanted forward lamina of L5 *(courtesy of Dr. Sarah Pownder, DACVR)*. **(D)** Same dog as in A, 3D reconstruction of CT allows to cut out anatomical areas to help visualize the path of the MC/MMC for surgical planning.

CT is well-suited for the investigation of NTDs as it allows for a fast examination, doable under sedation-only in a juvenile brachycephalic population, with imaging of the whole body to help in the diagnosis of further malformations. CT is ideal for osseous structures (e.g., spina bifida) and allows 3D reconstruction helpful in visualization of the path of the MC/MMC for surgical planning, with better spatial resolution than MRI (Figure 10). The most common main site of spina bifida for LS MCs/MMCs in our cohort was the last lumbar vertebra in 6/9 cases (5/9 were L7 and 1/9 was L8 due to further malformation) (see Figure 9). Other sites were L6 (1/9), and the sacrum (2/9, 1 affecting only S1, 1 affecting the entire sacrum). Three cases presented spina bifida at 2 or 3 adjacent sites (one at L6-L7, one at L8-S1, one over the entire sacrum) and in 4/9 the first spinous process and lamina directly cranial to the MC/MMC were abnormally shaped (caudal aspect of the lamina oriented dorsally and midline point of the lamina/base of the spinous process appearing shifted cranially due to the MC/MMC) (see Figure 10). Literature cases report specifically the site of spina bifida in 8/14 cases with the sacrum being the most common site (5/8), followed by the last lumbar vertebra L7 (3/8, with 1/3 being L7 + S1) (13, 15–19). In one cohort of 6 dogs in the literature, spina bifida was reported consistently at an intervertebral space/two consecutive vertebrae, either L6-L7 (3/6) or L7-S1 (3/6) (14).

##### Concurrent, separated sites of spina bifida (caudal vertebrae)

In all cases of the authors experience in Bulldogs (9/9), and in all literature surgical cases and descriptive reports where images of the caudal vertebrae were available for review (16, 18, 21, 32), it is noticeable that SB of the caudal vertebrae was present too, whether documented or not (see Figure 10). This is likely the consequence of primary neurulation failure at the caudal neuropore (site of the MC/MMC), hence preventing a normal secondary neurulation, although the mechanism may be specific to screw-tail breeds considering literature report (15) and personal experience of the authors with normal-tailed animals with sacral MCs/MMCs. Anecdotally, the authors have encountered mild/asymptomatic forms at T1-T2 (see Figure 2), as reported previously in Pugs (39, 40).

#### Magnetic resonance imaging (MRI)

**Figure 11:**
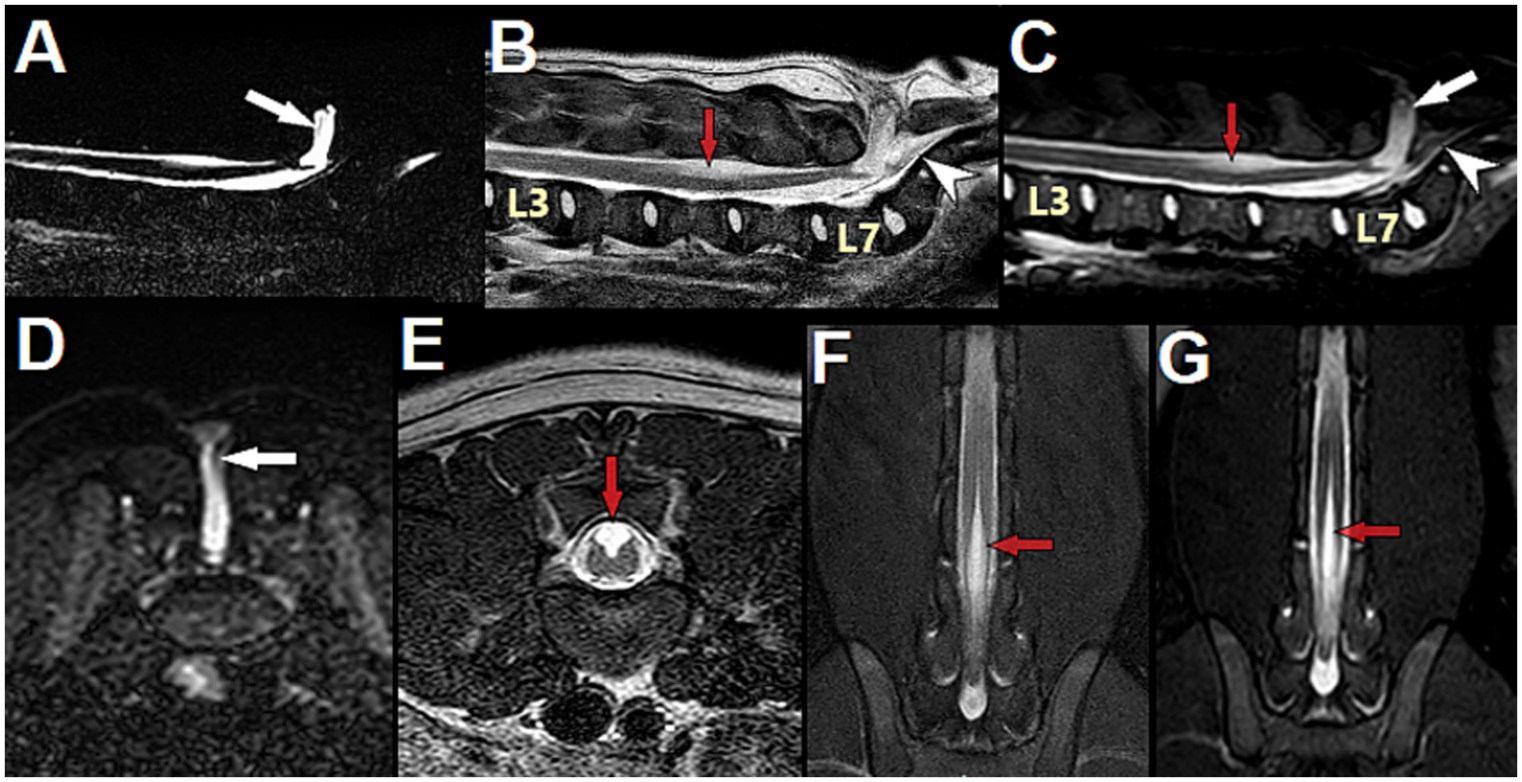
3-month-old male English Bulldog with myelomeningocele (MMC) and spina bifida (SB) over the sacrum, magnetic resonance (MR) images. **(A)** Sagittal MYELO, **(B)** Sagittal T2, **(C)** Sagittal STIR, **(D)** Transverse STIR at S1, **(E)** Transverse T2 at L5, **(F)** Dorsal T2 mDIXON and **(G)** Dorsal STIR at the level of the dorsal horns. Note the abnormal structure present in the MMC and tethering the conus medullaris to the distal end of the MMC (thin vertical grey line in **(A,C,D)** *white arrow*). On sagittal images, the conus medullaris and filum terminale internum are present in the normal dural sac, underneath the MMC, while the filum terminale externum is seen caudal to the MMC (hypointense horizontal line in the vertebral canal at the level of the sacrum, *white arrowhead*). Note the central cord hyperintensity suggestive of hydromyelia at L3, and the area of dorsal hyperintensity associated with the unfused dorsal cord at L5 (*red arrow*). Note the open dorsal median sulcus **(E–G)** and the peculiar aspect of the unfused dorsal spinal cord on transverse view **(E)**, showing the failure of midline fusion of the alar plate characteristic of spinal dysraphism (different than syringomyelia).

MRI is the imaging diagnostic modality of choice to visualize the presence or lack thereof of nervous element in the MC/MMC, the position of the spinal cord and the conus medullaris both within the vertebral canal and the dural sac, and any associated spinal cord changes. Since they contain CSF +/− nervous structures, MCs/MMCs appear as T2W and STIR hyperintense, T1W and FLAIR hypointense structures continuous with the normal dural sac, protruding through the vertebral canal and directed towards the skin (Figure 11). MRI allows for best visualization of the nervous tissue within the MC/MMC prior to surgery, although in the authors’ experience, the intra-operative findings of nervous tissue components within the MC/MMC do not always match with imaging-based expectations (obtained with a 3 T MRI). In our cohort 1 dog presented a MC, 8 dogs MMCs (with 1/8 presenting a lipomyelomeningocele [the distal end of the MMC including a lipoma], see Figure 6).

On MRI, tethered cord syndrome was defined as dorsal deviation of the spinal cord and was present in 9/9 cases in our group and in all 9 cases in the literature where that information was available (in 5/14 cases the information regarding dorsal displacement or not of the conus medullaris on imaging was lacking). Since both the anatomical malformations of the MC/MMC and the deviation of the nervous structures are obvious on static images in our experience, no dynamic views were performed in our group; none are reported in the literature either.

The conus medullaris position on MRI was L7 in 7/9 cases (4/7 were in the MMC, 3/7 were ventral to the MMC and still in the vertebral canal) and L6 in 2/9 cases (with the MMC containing nerves but no spinal cord). It was documented in only 4 cases in the literature, all reporting it in the MMC at surgery (sacrum for 3 cases, L7 for 1). The filum terminale internum is in our experience most commonly at the base/bottom of the MC/MMC without being in it, while the filum terminal externum is usually caudal to it, in the vertebral canal at the level of the sacrum (see Figure 11).

**Figure 12:**
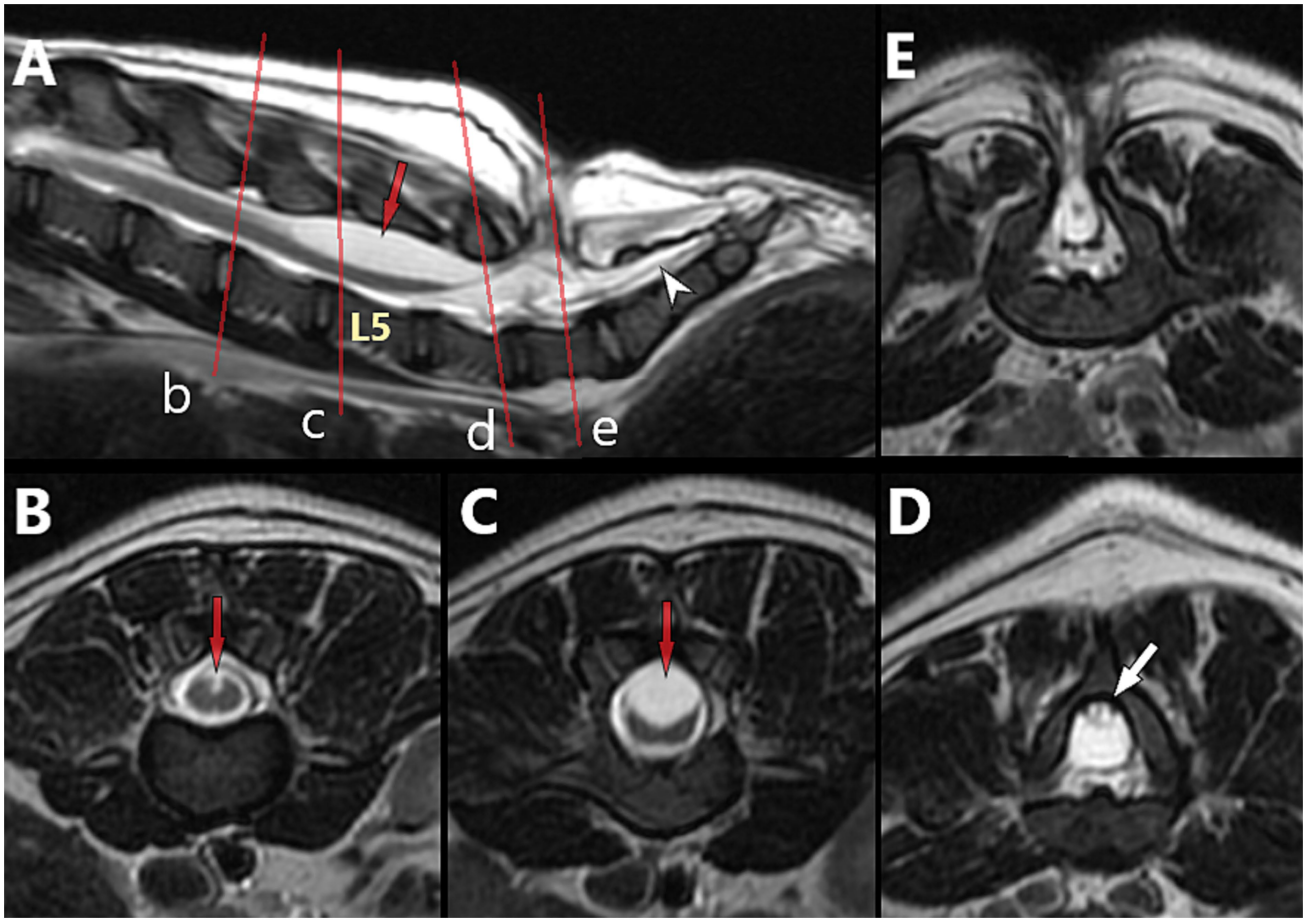
5-month-old male French Bulldog with myelomeningocele (MMC) and spina bifida (SB) at L7 (dog B in Figure 7), T2-weighted magnetic resonance (MR) images. **(A)** Sagittal view with indications of the level of the transverse images (*red lines*) at L4 **(B)**, L5 **(C)**, caudal L6 **(D)** and into the MMC at L7 **(E)**. Note the characteristics of neural tube defect (NTD)/spinal cord dysraphism with the unfused dorsal median sulcus over L5 (*red arrow*), and the TCS with dorsally deviated dural sac and conus medullaris at L6 (tucked against the lamina, *white arrow*), suggestive of adhesions between the dorsal aspect of the dural sac and the ventral aspect of the lamina of L6 (see Figures 14C–E for intra-operative images).

Further changes in the nervous tissue, signs of TCS and NTD/spinal cord dysraphism, were identified in our cohort such as: *syringohydromyelia* in 5/9 cases (sometimes all the way to the cervical spinal cord), signs of Chiari-like malformation (CLM) regarding the position of the cerebellum and ventriculomegaly/hydrocephalus in, respectively, 2/6 and 5/6 cases where MRI of the brain was performed (see Figure 6), and an open dorsal median sulcus/failure of fusion of the spinal cord on dorsal midline in all MMCs (8/9) (although only minimal/mild in 2 of the 8) (see Figures 11, 12). This last finding correlates with previous reports of structural changes to the dorsal midline of the spinal cord on histology: lack of dorsal median septum, disruption of the ependyma with elongation of the central canal in a slit-like cavitation in the dorsal funiculus (32), and eventually obliteration of the later resulting in an “open” cord covered dorsally solely by a remnant of pia matter (11). This may be difficult to differentiate from *syringomyelia* on MRI as both appear as intramedullary T2W hyperintense, T1W hypointense and FLAIR suppressing areas (although the transverse appearance is sometimes unequivocal – see Figure 11E), and we cannot ascertain that the separate sites of syringomyelia seen in 5/9 of our cases and attributed to TCS (all 5 cases presented worsening of signs) did not represent several sites of failure of proper formation of the neural tube, since NTDs frequently affect multiple areas. If so, the term *hydromyelia* originally defined as dilation of the central canal might be more appropriate and anatomically accurate, since NTDs imply an abnormal/improperly closed central canal (the term *syringohydromyelia* indicates uncertainty regarding communication with the central canal) (see Table 1). Although the term syringomyelia was used in previous reports featuring similar images of unfused/split cord on dorsal midline usually cranial to the MMC (14, 20), it is our impression that these changes are reflective of the nature of NTDs, with a lack of fusion of the 2 dorsal hemi-cords on midline, rather than true syringomyelia (i.e., acquired pathology whereby a syrinx develops in the spinal cord parenchyma without communication with the central canal), which can be seen as a result of TCS.

None of the cases in our cohort showed a true dermoid sinus associated with the MC/MMC. A series of 3 cases report dermoid sinus but no imaging details were available for review (19). Despite the term being used in the literature, there is no detailed advanced imaging or histological report of a true dermoid sinus associated with a LS MC/MMC in dogs to our knowledge thus far, although dermoid sinus have been reported in other areas of the neuraxis, including the head (e.g., nose) (see Discussion).

### Treatment options and prognosis

#### Medical management

##### Treatment

Medical management for LS MCs/MMCs is limited to palliative care regarding the various pathologies and complications associated: management of urinary tract infections, possible rectal prolapses, dermatopathies associated with urinary/fecal incontinence, diet adjustments to help manage fecal incontinence (aiming for low-volume of formed feces), management of neurological deficits (e.g., use of cart to help with pelvic limbs mobility) and associated skin excoriations. Pain is not a predominant feature of this disorder in our experience, but anti-inflammatory for local discomfort at the MC/MMC site and gabapentin +/− amantadine for neuropathic pain may be warranted. Antibiotics may be warranted for cases of open MC/MMC (in case of ascending infection).

##### Outcome

There is a paucity of reports of long-term results of medical management of dogs with LS MC/MMC in the literature, with information being limited to the history until presentation since most dogs were euthanized in early studies (11, 32). Although occasionally described and thought of as static, reports of LS MC/MMC in the literature also describe possible progressive worsening of neurological deficits in the absence of surgical correction (21, 38). This has been our experience, with personal communication with volunteers in Bulldog rescue organizations reporting progressive worsening of paraparesis and para-ataxia over several months/year in some dogs not corrected surgically, although a plateau of neurological deficits where things remain static is most often reached. The continence may also worsen, with some cases suffering initially from only partial/occasional urinary or fecal incontinence progressing to complete urinary and fecal incontinence over time. This also fits with the finding of 7/9 dogs presenting with worsening clinical signs and clinical TCS in our study. While dogs with only minimal, non-progressive neurological deficits may not warrant specific treatment, animals more affected do.

#### Surgical management

In general, the goals of surgery for MC/MMC should be to:

- Reestablish normal position of the nervous structures in the vertebral canal.
- Untether the nervous structures and meninges:
- Remove adhesions of the dural sac with periosteum of adjacent laminae.
- Remove adhesions of the nervous structures (within the dural sac) with arachnoid/dura if present.
- Close the dural defect (for open MC/MMC).

##### Procedure

**Figure 13:**
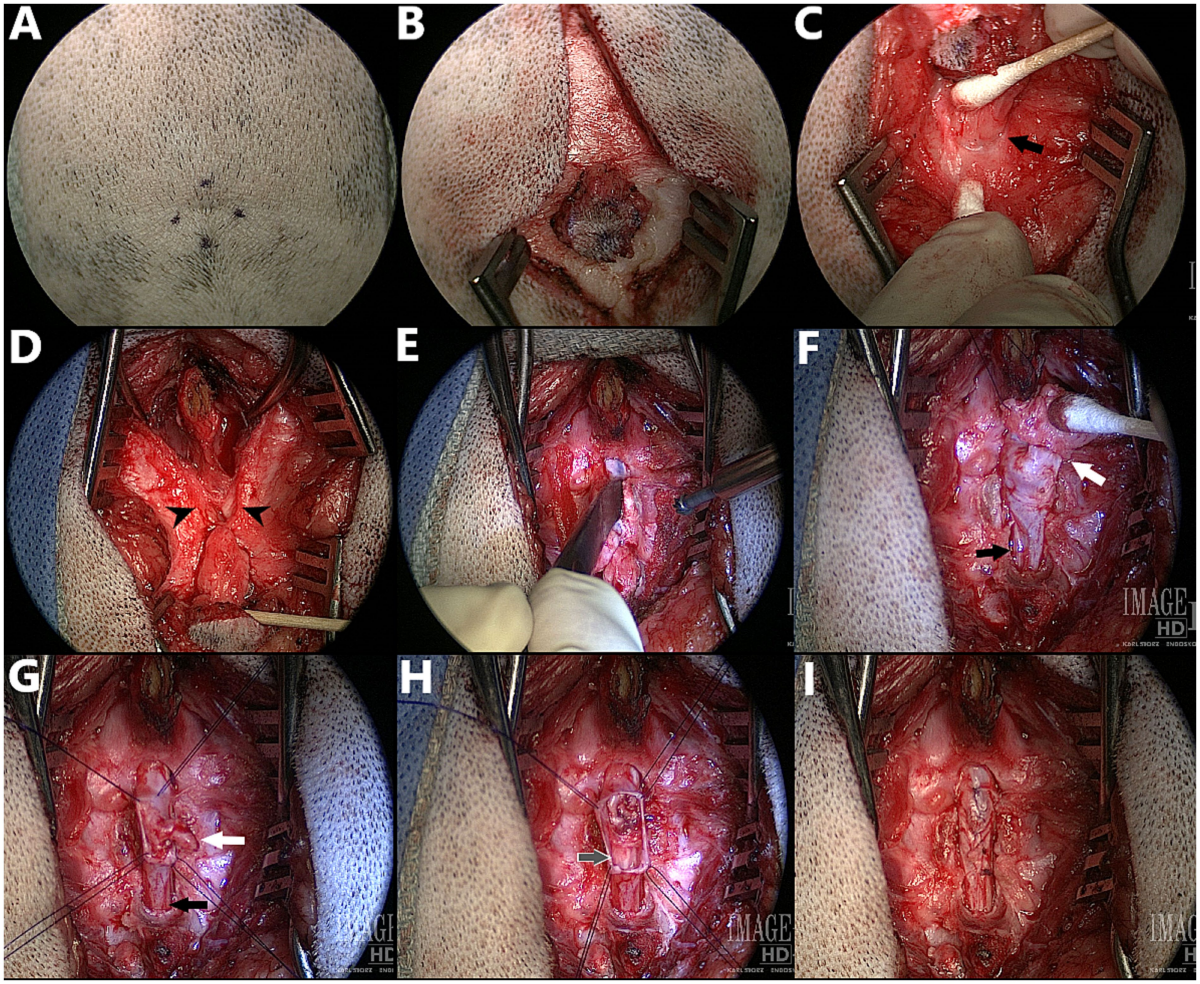
Surgical correction of myelomeningocele (MMC) with spina bifida (SB) at L6 in a 3.5 month old male English Bulldog, dorsal view – cranial at the top of the images (Dog D in Figure 7, radiographs in Figures 9A,B, CT in Figure 10C). **(A,B)** Cutaneous incision. **(C)** Dissection around the abnormal fibroadipose tissue (*black arrow* indicates the palpable dorsal midline defect in the lumbar muscles). **(D)** Incision and elevation of the lumbar muscles (*black arrowheads* indicate the two unfused hemi-spinous processes at the site of SB). **(E)** Laminectomy at the site of SB +/− adjacent sites if required for visualization of the MMC. **(F)** Visualization of the MMC, reclined cranially for presentation (*white arrow* indicates the attachment of the fibroadipose tissue onto the distal end of the MMC, *black arrow* indicates normal dural sac caudal to the MMC). **(G)** Durectomy performed (*white arrow* indicates the aberrant nervous structure terminating as a dead-end in the MMC that is resected, *black arrow* indicates the caudal dural sac and continuation as filum terminale externum). **(H)** Durectomy performed (continued) (*grey arrow* indicates the sacrocaudal nerves and filum terminale internum caudal to the MMC; visualized discoloration due to use of Surgicel®). **(I)** Closure of the dura with simple continuous pattern (6–0 Vicryl™) before routine closure.

The surgical procedure performed by the author is described below and imaged step-by-step in Figure 13.

**Figure 14:**
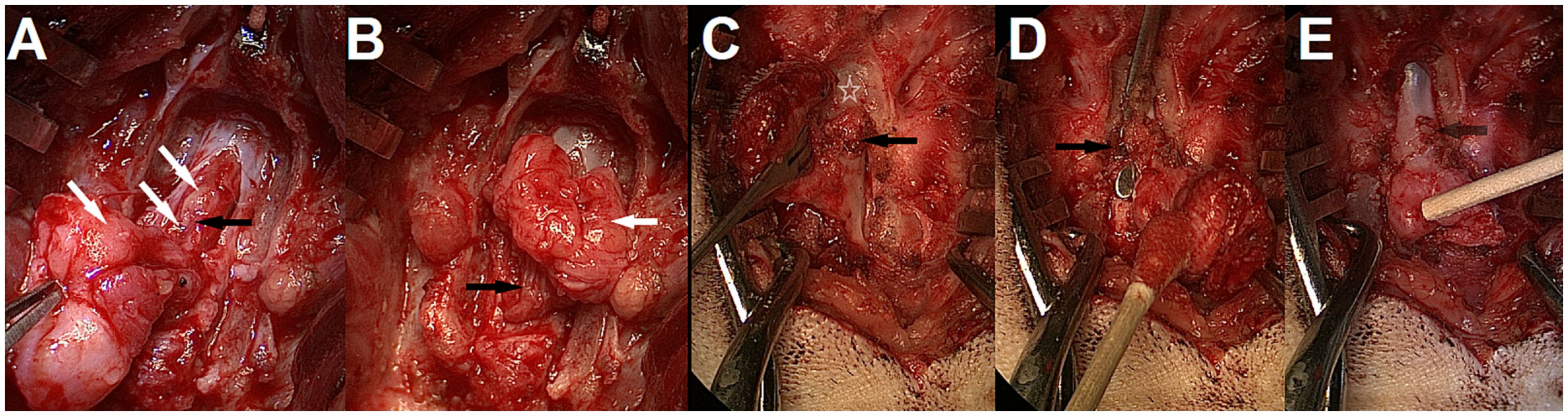
Intra-operative findings and adhesions. **(A,B)** Lipomyelomeningocele in a 3-month-old male French bulldog/pug mix with spina bifida (SB) at L7 (MRI in Figure 6). Laminectomy of L6, removal of the bifid spinous processes of L7 and durotomy were performed. Note the cauda equina infiltration by the lipoma (*white arrows*). The sacrocaudal nerves are visible in the lipomatous mass and caudal to it after partial dissection (*black arrows*); they should be preserved. **(C–E)** 5-month-old male French Bulldog with MMC and SB at L7 (dog B in Figure 7, MRI in Figure 12) **(C)** Partial laminectomy of L6 (*white star*), showing thick fibrous adhesions (*black arrow*) between the ventrocaudal aspect of the lamina and the dura/MMC. **(D)** Laminectomy performed at L6 with spatula between the dural sac and adhesions (*black arrow*) for visualization. **(E)** Removal of adhesions complete, the attachment point can be seen (*see-through black arrow*).

The patient is clipped and aseptically prepared for surgery on dorsal midline from ~L3 to the tailbase, over a width slightly larger than the wings of the ilium. If fecal leakage is significant, a purse-string suture can be used for the time of the surgery. Positioning is in sternal recumbency, with pelvic limbs forward and support (e.g., surgical towels, bean bag) under the pelvis to maintain stability. Cutaneous incision starts with a round or elliptical incision centered on the swirl of hair and is extended on sagittal midline cranially and caudally (Figures 13A,B). The subcutaneous and fat tissues are dissected until a cylindrical structure underlying the area of deepest cutaneous depression can be identified. Dissection is continued along the stalk of abnormal fibroadipose tissue as it “dives” between the lumbar muscles and reaches the lumbodorsal fascia, using a combination of blunt and sharp dissection (cotton tip applicators can be used to “brush off” the softest fibrous tissues from the stalk, Castroviejo, iris and corneal scissors for sharp dissection of the toughest tissues) (Figure 13C). The change in orientation of the lumbodorsal fascia fibers mark the dorsal midline defect (*black arrow* in Figure 13C) through which the MC/MMC passes and can be used as landmark to palpate the two unfused spinous processes at the site of spina bifida. The lumbodorsal fascia is then incised around the first normal spinous process just cranial to the defect (e.g., L6 if spina bifida at L7) and the incision is continued caudally around the MC/MMC on each side of the SB site (Figure 13D). The multifidus lumborum (cranially) and sacrocaudalis dorsalis medialis (caudally) muscles are elevated from the first normal spinous process, the two unfused hemi-spinous processes at the site of SB (*black arrowheads* in Figure 13D) and the median sacral crest, and are maintained with Weitlaner and/or Gelpi retractors. In our experience, further attachment points of the MC/MMC to adjacent structures are common (e.g., on dorsal midline, to the ventro-caudal aspect of the dorsal lamina of the normal vertebra just cranial/caudal to the MC/MMC; on lateral aspects, to the hemi-spinous processes at the site of SB). If an attachment point to the lamina of the vertebra cranial (or caudal) to the site of SB was seen on images or at surgery, a partial laminectomy of this vertebra should be performed to remove all adhesions and ensure that no tethering persists. A combination of bipolar cautery on very low setting, Castroviejo scissors and number 11 blade is used to remove all adhesions between MC/MMC and surrounding vertebrae and ligamentous structures (note the difference between E and D, also see Figure 14). The unfused hemi-spinous processes may need to be removed to facilitate manipulation of the dural sac (Figure 13E). This can be done with rongeurs or high-speed drill if the cord is protected by a flat and blunt instrument on the medial aspect of the bone. The stalk of abnormal fibrous tissue and skin covering the distal end of the MC/MMC can be dissected from it using Castroviejo, iris or corneal scissors once a cleavage plan is identified (*white arrow* in Figure 13F), working from normal dura at the SB site towards the abnormal tissue. The end of the dural sac is usually covered by this abnormal tissue and extends deeper into it than initially visible (similar to a mushroom cap covering the stem). The dissection continues in a degloving pattern and is finished circumferentially at the most distal end of the MC/MMC with transection of any “dead-end” malformed nervous structure ending in the skin. The part of the normal dural sac not involved in the MC/MMC and caudal to it (*black arrow* in Figure 13F), including the filum terminale, should be visualized and checked for adhesions. Once the dural sac is freed from all attachments around the MC/MMC, 4 stay sutures are placed at the corners of the MC/MMC prior to performing a circumferential durotomy at the base of it (Figure 13G). The dura of the MMC is progressively everted in degloving pattern to ensure that arachnoid adhesions attaching nervous structures (nerve roots) to the abnormal dura are incised progressively. This is continued until the distal end of the MMC, where any “dead-end” structure (*white arrow* in Figure 13G) finishing in or through the dura should be excised, while any structure folding around to go back down into the caudal dural sac (*black arrow* in Figure 13G) may represent a viable sacrocaudal nerve and should be preserved. Once the abnormal dura is removed, the preserved nervous structures are replaced in the underlying normal dural sac (*grey arrow* in Figure 13H) which should be inspected for further adhesions before closure. The dura is closed with a simple continuous pattern (6–0 Vicryl™) (Figure 13I). A fat pad or Gelfoam® layer is placed over the laminectomy defect and remaining closure is routine.

##### Intra-operative findings

In our cohort, 1/9 was a MC and 8/9 were MMCs, with 1 of the 8 MMCs presenting a large lipomatous mass at the distal end of the dural sac, in and through the MMC, continuous from the intradural compartment to the subcutaneous tissue (through the meninges), prompting a diagnosis of lipomyelomeningocele (transitional/chaotic lipoma if applying human classification) (Figure 14). Numerous leptomeningeal adhesions between the nerve roots of the cauda equina and the lipoma or dura of the MMC had to be dissected for progressive lipoma removal.

One interesting intra-operative finding was the presence of adhesions between the dural sac/MC/MMC and the surrounding structures (ventral lamina of the normal vertebrae adjacent to the MC/MMC [e.g. L6 or S1 if SB at L7], bifid spinous process, ligamentous structures). Although not documented systematically in our first cases, this seems to be the rule more than the exception in Bulldogs and was responsible for tethering of the cord (visualized by ascending movement of the dural sac in the vertebral canal upon removal) (see Figure 14). There were also occasional adhesions within the dural sac/MMC between the nervous structures and meninges, as reported before (14, 17).

##### Outcome

The dog diagnosed with lipomyelomeningocele died within 72 h of surgery (found acutely dead with notes documenting bright, alert and responsive (BAR) status <10 min before), leaving 8/9 dogs with long-term follow-up in our cohort (>6 months). Autopsy showed changes in the lungs with pulmonary edema and positive oil red O stain for lipid, prompting a presumptive diagnosis of pulmonary embolism of fat-containing tissue (e.g., lipoma, subcutaneous fat, bone marrow) (see Discussion). Out of the literature cases, 1 dog was euthanized at 2 weeks post-operatively with only minimal improvement reported (16) so it is not counted in outcome (timeframe too short to judge of surgical success or not in our experience). One dog died of parvovirus infection after 1 month post-operatively but had fully recovered continence and improved pelvic limbs deficits at day 15 (14), so is counted in outcome. All other literature cases had ≥3 months follow-up.

Regarding neurological deficits, our cohort and literature review document improvement of pelvic limb deficits, respectively, in 6/8 and 8/9 (total 14/17, ~82%) of dogs that had deficits before surgery and follow-up for ≥1 month. It should be noted however that this criterion can be vague and not the most relevant clinically, as motor and sensory deficits are mild to lesser in most cases and rarely the primary concern of the owners, the urinary/fecal incontinence is.

Recovery of urinary and fecal continence can be difficult to accurately report on, as many dogs do not have an all or nothing response, but a partial/incomplete recovery. The criteria we retain in evaluation of our population was to ask the owners if the urinary/fecal incontinence still warranted for the patient to wear diapers at home most of the time or was manageable without (i.e., does the pet still have to practically be managed for incontinence at home). As an example, a dog who only shows occasional feces dropping when running outside with no other sign at home would be considered having recovered. Regarding this criteria, review of our cohort and the literature shows recovery of urinary/fecal continence in, respectively, 4/8 and 6/13 dogs (total 10/21, ~48%). One dog in each group recovered/improved urinary or fecal continence but not the other so the 2 dogs were counted as non-recovery. In the total of 10/21 dogs that recovered, 1 of them died at 1 month of parvovirus infection (14) and 1 of our cohort lost urinary continence again due to a multi-drug resistant *Enterococcus faecium* urinary tract infection (UTI) with penile necrosis at ~6 month post-operatively (penile amputation and scrotal urethrostomy had to be performed), leaving a total of 8/21 (38%) dogs that remain alive and continent at 6 months post-operative.

## Discussion

The exact nature of the fibrous tissue between the distal dural end of the MC/MMC and the skin is unclear to our knowledge. Although dermoid sinuses have been documented histologically in MCs/MMCs of other areas of the vertebral column (41), it is not our experience that dogs with LS MCs/MMCs have a true dermoid sinus associated. The term was mentioned in a previous surgical report, with the authors taking the precaution to report a “dermoid sinus-like lesion” since no lumen was reported (18). All types of dermoid sinus, including suggested type VI, comprise at least in the superficial part a tubular structure with findings of follicular annexes and/or sebum in its lumen at surgery (42, 43). This is not our surgical experience. Although no histological analysis was performed in our cohort, gross macroscopic impression is the one of abnormal fibrous & fatty (fibroadipose) tissue, without any tubular, hollow structure required for the qualification of dermoid sinus. It is possible that the appearance of the sagittal views on CT/MRI images with the skin creating a dimple/well at the attachment point of the MC/MMC (see Figures 10, 12) has led to the erroneous use of that term, along with the appearance of the cylindric fibrotic structure connecting the MC/MMC to the skin (since the surgical act of dissecting along a hollow tube or a plain cylinder is similar). It is also possible that this abnormal tissue constitutes a new subtype of dermoid sinus devoid of lumen and adnexal structures entirely, but this is unreported and contrary to the current definition of dermoid sinus. Published reports with autopsy/histopathology available do not describe a dermoid sinus (32) or lack description of skin changes (11). One series of 3 dogs mention dermoid sinus but no specific information on images or histology could be retrieved, and no mention of any hollow structure containing adnexal structure/sebum is made (19).

### Post-operative complications and outcome

Review of our cases and literature reports 8/9 + 14/14 (22/23, ~96%) dogs that underwent surgery and recovered without major neurological worsening, although 1 dog in our cohort died <72 h after surgery. Considering the presumptive diagnosis of fatty pulmonary embolism (see Treatment/Surgical management/Outcome) and the extensive dissection of a lipomatous mass that was required at surgery (see Figures 14A,B), this should be considered a possible complication for surgical repair of lipomyelomenigocele (although the origin of the fatty embolism cannot be ascertained, a bone marrow origin seems less likely in a juvenile patient). Temporary neurological worsening was observed in the immediate post-operative period (<48 h) in 1/9 dogs in our cohort and 2/14 in the literature (14), all back to pre-operative status or improved after 48 h. Regardless of other unrelated death/complications, no long-term neurological worsening has been reported thus far, hence the surgery appears overall safe from a neurological standpoint.

Although there is a lack of medically treated control group to compare, the recovery rates of ~48% for urinary/fecal incontinence and ~ 82% for pelvic limbs deficits appear favorable to recommending surgical management in most cases. It remains unclear if earlier surgery would be associated with a better functional and overall outcome. On one hand, human studies have clearly shown the benefits of earlier intervention in development, leading to the current consensus of intra-utero surgery since the Management of Myelomeningocele Study (MOMS trial) (7); on the other hand, in-utero surgery being unrealistic in veterinary medicine in the near future, one must consider the risks involved with anesthetizing the typical patient, already brachycephalic and juvenile, at an even younger age.

Regarding the recovery of urinary/fecal continence, we have not been able to isolate a possible influence of age at surgery, severity of symptoms, nor imaging changes (such as position of the conus medullaris). Although reported in terms of continence vs. incontinence, the post-operative urinary/fecal functions rarely fall within a purely dichotomic classification of normal vs. abnormal. Several dogs considered to remain urinary/fecal incontinent did show signs suggestive of an improvement of the urinary function, such as easier bladder expressions, bladder found empty on recheck examinations and/or absence of UTIs post-operatively (12, 13, 16). Similarly, the multi-drug resistant *Enterococcus faecium* UTI with penile necrosis that led to scrotal urethrostomy and loss of regained urinary continence in one of our cases might be a sign of incomplete urinary recovery, since a similar case of penile necrosis was reported in a medically managed 5 month old Cocker Spaniel with MMC and SB at L7 (10).

The significance of the open dorsal median sulcus documented previously (11, 32) and in 8/9 patients on MRI in our cohort (see Figures 6, 11, 12) from a developmental, neuro-anatomical and recovery standpoint is unclear. It is possible that this sign of NTD/spinal dysraphism be accompanied with further anomalies of neuronal migration and neuronal architecture of the spinal cord, which might preclude recovery even after gross anatomical correction (i.e., this aspect of the pathophysiology of LS MCs/MMCs may be more relevant for continence prognosis than the TCS). Although small numbers do not allow to draw conclusion, the 1/9 dog who did not present this finding (the only MC) and 2/9 that were qualified of only minimal/mild changes were all within the 4 dogs of our cohort who had the best recovery (both pelvic limbs function and continence).

### Surgical technique

The surgical technique utilized by the authors and the ones described in the literature are sensibly similar with a common dorsal midline approach, dissection around the tract of the MC/MMC followed by durotomy/durectomy, transection +/− removal of abhorrent structures and closure (12–19). Several questions regarding best surgical options persist. The adhesions found between the dural sac and adjacent structures (most commonly ventral lamina of the normal vertebra cranial to the MC/MMC) were responsible for tethering, so we believe they should be identified on images and addressed surgically with partial laminectomy of affected vertebrae. Interestingly, we have not made the same observation in cases of feline LS NTDs that we treated surgically. Intradural adhesions between meninges and nerve roots present in the MMC also require dissection as reported before (14), which can be surgically challenging; this phenomenon was most severe in the case of lipomyelomeningocele in our experience (see Figure 14).

Re-tethering is a documented complication in human LS MMC surgical repair and, although it has not yet been described in veterinary medicine, there is no report of imaging follow-up post-operatively in dogs aside of a partial one at 8 weeks (20). Filum terminale transection has been used successfully to treat TCS in humans after LS MMC surgery and in veterinary patients with occult TCS (28–30). The role of the filum terminale in the pathophysiology of canine LS MMC, hence the surgical attitude to employ, are unclear. Surgical practices in humans focus on transection of the filum terminale internum (FTI) following durotomy, with authors arguing that transection of the filum terminale externum (FTE) is unlikely to have significant effect on TCS due to lack of impact of FTE tension on the position and morphology of FTI (44). By opposition, all reported cases of occult TCS in the veterinary literature and cases treated successfully by the authors all underwent transection of the FTE only (28–30). Filum terminale transection was mentioned in several literature cases of MC/MMC (13, 14, 16), however surgical reports describe the transection of the abnormal cul-de-sac structure present in the MMC rather than true section of the FTI (in our experience not located in the vertical part of the MMC, but in the dural sac present at the base of the MMC or caudal to it) or FTE (usually present caudal to the MMC, in the vertebral canal of the sacrum, and requires laminectomy of the sacrum for visualization) (see Figures 11–13).

### Limitations

This review has several limitations. First, only the English literature was reviewed, so reports in other languages may have been ignored. Second, although the authors included every dog treated surgically until the redaction of this manuscript (i.e., no dog who underwent surgery was excluded), the overrepresentation of case reports and very small case series in the literature introduces the risk for several biases: selection bias, lack of consecutive sampling (aside of our cohort), lack of prospective data collection in favor of retrospective description, and the absence of control group. Other limitations include the lack of imaging follow-up (preventing conclusion regarding restoration of normal anatomy and evolution of the dysraphic area of nervous tissue), the lack of histological analysis of the cul-de-sac nervous structure resected at surgery (preventing conclusion regarding the exact nature of this tissue), and the lack of neurophysiological assessment through electrodiagnostics.

Intra-operative neuromonitoring of human patients undergoing tethered cord release after having undergone intra-utero MMC closure (as fetuses) confirmed a motor-sensory discordance with altered somatosensory pathways (45). This is likely related to the pathophysiology of NTDs (affecting specifically the dorsal part of the neural tube) and reflective of further disturbances in the formation of the alar plate, which might present a challenge for recovery of continence. In humans, urodynamic testing can help predict some complications such as upper urinary tract changes related to myelodysplasia (46), while EMG of SB patients has shown changes in the tibialis anterior, extensor digiti brevi, gastrocnemius medialis and external anal sphincter (the latter being significantly more affected in *spina bifida aperta*) (47). Needle EMG of the external anal sphincter and the tail base muscles of a 2-month-old English Bulldog who did not recover continence post-surgery showed absence of any insertion potential or spontaneous activity (13). EMG of the pelvic limbs was normal in this dog and in 1 out of 2 bulldogs treated with mesenchymal stromal cells; it showed mild changes in the other, while both sensory and motor nerve conduction velocities were within normal limits (20). EMG of the external anal sphincter and somatosensory testing might represent prognostic indicators and this should be evaluated in future canine studies.

## Summary and conclusion

Lumbosacral MCs and MMCs are congenital malformations affecting preferentially screw-tail breeds (e.g., Bulldogs), often associated with tethered cord syndrome and potential worsening of neurological deficits at a young age. Due to the specific pathophysiology of neural tube defects, these patients present a constellation of neurological deficits (urinary/fecal incontinence, bunny hopping, pelvic limbs deficits) and external signs (e.g., swirl of hair with dimple in the skin +/− lumbar muscle) that is pathognomonic of their condition. Ideal imaging recommendation for surgical planning is, in order, MRI + CT > MRI > CT. Other co-malformations, such as orthopedic issues and cryptorchidism are common. Surgical correction can be challenging, specifically in cases of lipomyelomeningocele, but appears overall safe with 22/23 dogs surviving the post-operative period and no case of major worsening yet reported. While the results might be biased positively due to the absence of prospective study and to the hegemony of case reports and small case series, post-operative neurological improvement/recovery is possible, although it is less frequent for urinary and fecal continence (<50%) than for pelvic limbs deficits (~80%). Future studies are required to identify prognosis regarding recovery of fecal and urinary continence. It is recommended to address adhesions of the dural sac with adjacent bones (usually ventral aspect of the lamina) and leptomeningeal adhesions within the dural sac during surgery, in an effort to release the tethered nervous structures.
